# Supplementary material for: Salmonella Vaccine Study in Oxford (SALVO) trial: protocol for an observer-participant blind randomised placebo-controlled trial of the iNTS-GMMA vaccine within a European cohort
Source: BMJ Open. 2023 Nov 14;13(11):e072938. doi: 10.1136/bmjopen-2023-072938 (PMC10649500; doi:10.1136/bmjopen-2023-072938)
Supplement: Supplementary data [file bmjopen-2023-072938supp001.pdf]

Trial Title: A Phase 1 Clinical Study to Determine the Safety and Immunogenicity of a Novel GMMA Vaccine Against Invasive Non-Typhoid Salmonella

Internal Reference Number / Short title: OVG2020/01 Salmonella **V**accine Study in **O**xford (SALVO)
Ethics Ref: 22/SC/0059
IRAS Project ID: 1005098
EudraCT Number: 2020-000510-14
Date and Version No: 13 January 2023 / Version 3.1

Chief Investigator: Dr Maheshi Ramasamy
Oxford Vaccine Group (OVG)
Department of Paediatrics
University of Oxford
Centre for Clinical Vaccinology & Tropical Medicine (CCVTM)
Churchill Hospital
Oxford
OX3 7LE
United Kingdom

Co-Investigators: Professor Andrew J Pollard

Dr Brama Hanumunthadu

Sponsor: University of Oxford
Funder: EU Framework Programme for Research and Innovation, Horizon2020, Vacc-iNTS
The GSK Vaccines Institute for Global Health (Monitoring Costs Only)

Chief Investigator Signature:

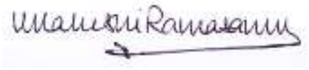

Statistician Signature:

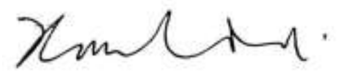

We declare no financial conflict of interest in this study.

Confidentiality Statement
This document contains confidential information that must not be disclosed to anyone other than the Sponsor, the Investigator Team, HRA, host organisation, and members of the Research Ethics Committee and Regulatory Authorities unless authorised to do so.

Date and version No: 13January2023 Version 3.1

TABLE OF CONTENTS

1 KEY TRIAL CONTACTS 7
2 LAY SUMMARY 8
3 SYNOPSIS 9
4 ABBREVIATIONS 13
5 BACKGROUND AND RATIONALE 15
5.1 INVASIVE NON-TYPHOIDAL SALMONELLA DISEASE 15
5.2 BURDEN OF DISEASE 15
5.3 IMMUNO-PATHOGENESIS 16
5.4 PRIOR EXPERIENCE WITH iNTS VACCINES 17
5.5 RATIONALE FOR VACCINE BASED ON GMMA TECHNOLOGY 17
5.6 CLINICAL STUDIES OF ENTERIC GMMA VACCINES 18
5.7 DESCRIPTION OF INVESTIGATIONAL PRODUCT 19
5.8 CHARACTERISTICS OF VACCINE PREPARATION 19
5.9 IMMUNOGENICITY IN MICE 19
5.10 IMMUNOGENICITY IN RABBITS 20
5.11 TOXICOLOGY 20
5.12 RATIONALE / AIM OF TRIAL 22
6 OBJECTIVES AND OUTCOME MEASURES 23
6.1 PRIMARY OBJECTIVE 23
6.2 SECONDARY OBJECTIVE 23
6.3 EXPLORATORY OBJECTIVES 23
6.4 PRIMARY ENDPOINTS / OUTCOME MEASURES 23
6.5 SECONDARY ENDPOINTS / OUTCOME MEASURES 24
6.6 EXPLORATORY ENDPOINTS / OUTCOME MEASURES 24
7 TRIAL DESIGN 25
7.1 OVERVIEW OF TRIAL DESIGN 25
7.2 DOSE ESCALATION 26
7.2.1 DOSE ESCALATION PROCESS FOLLOWING D0 VACCINE 26
7.3 DSMC (Unblinded) REVIEWS 28
7.4 GROUP ALLOCATION 28
7.5 SAFETY MONITORING 28
8 PARTICIPANT IDENTIFICATION 33
8.1 TRIAL PARTICIPANTS 33
8.2 INCLUSION CRITERIA 33
8.3 EXCLUSION CRITERIA 33

CONFIDENTIAL

Hanumunthadu B, *et al. BMJ Open* 2023; 13:e072938. doi: 10.1136/bmjopen-2023-072938

8.4 TEMPORARY EXCLUSION CRITERIA ... 35
8.5 PREGNANCY AND CONTRACEPTION ... 35
9 TRIAL PROCEDURES ... 36
9.1 RECRUITMENT ... 36
9.2 INFORMED CONSENT ... 37
9.3 BASELINE ASSESSMENTS AT SCREENING ... 38
9.4 RANDOMISATION ... 39
9.5 BLINDING AND CODE-BREAKING ... 39
9.6 VACCINATION VISITS ... 39
9.7 NON-VACCINATION VISITS ... 40
9.8 OUTSIDE OF CCVTM VISITS ... 40
9.9 LABORATORY INVESTIGATIONS ... 40
9.9.1 ANALYSIS OF BACTERICIDAL ACTIVITY (GVGH) ... 41
9.9.2 FURTHER ANALYSIS OF FUNCTIONAL ANTIBODIES (OVG and Collaborators) ... 41
9.9.3 ANALYSIS OF ANTIBODY CONCENTRATIONS AGAINST O-ANTIGENS (GVGH) 41
9.9.4 ANALYSIS OF ANTIBODY CONCENTRATIONS AGAINST PORIN AND OTHER ANTIGENS (OVG and Collaborators) ... 41
9.9.5 CELLULAR RESPONSES AND CYTOKINE RELEASE (OVG and Collaborators) ... 41
9.9.6 ANALYSES OF B CELL RESPONSES (OVG and Collaborators) ... 41
9.9.7 ANALYSES OF T CELL RESPONSE AND CYTOKINE RELEASE (OVG and Collaborators) ... 42
9.9.8 ANALYSIS OF GENE EXPRESSION (OVG and Collaborators) ... 42
9.9.9 ANALYSIS OF GENETIC DETERMINANTS OF VACCINE RESPONSE (OVG and Collaborators) ... 42
9.9.10 ANALYSIS OF ORAL FLUID ANTIBODY CONCENTRATION AGAINST O ANTIGENS AND PORINS (OVG and Collaborators) ... 42
9.9.11 COLLECTION OF SERUM TO BE USED AS A REFERENCE STANDARD FOR THE SET-UP OF LABORATORY ASSAYS IN CURRENT / FUTURE STUDIES (OVG/GVGH and Collaborators) ... 42
9.9.12 ANALYSIS OF FAECAL ANTIBODY CONCENTRATION AGAINST O ANTIGENS (OVG and Collaborators) ... 43
9.9.13 INVESTIGATION OF IMPACT OF VACCINATION ON GUT MICROBIOTA (OVG and Collaborators) ... 43
9.10 SAFETY BLOOD TESTS ... 43
9.11 TRIAL PROCEDURE SCHEDULE ... 44
9.12 EARLY DISCONTINUATION / WITHDRAWAL OF PARTICIPANTS ... 44
9.13 DEFINITION OF END OF TRIAL ... 45

9.14 SPECIAL CIRCUMSTANCES: COVID-19 45
9.14.1 STUDY CONDUCT / RISK ASSESSMENT 45
9.14.2 COVID-19 INFECTION CONTROL MEASURES AT VISITS 45
9.14.3 PARTICIPANTS UNDER QUARANTINE 46
9.14.4 PARTICIPANTS WITH COVID-19 SYMPTOMS 46
9.14.5 PARTICIPANTS INVITED FOR COVID-19 VACCINATION DURING THE TRIAL 46
10 TRIAL INTERVENTIONS 47
10.1 INVESTIGATIONAL MEDICINAL PRODUCT (IMP) DESCRIPTION 47
10.1.1 iNTS GMMA VACCINE 47
10.1.2 PLACEBO 47
10.2 BLINDING OF IMPS 47
10.3 STORAGE OF IMP 47
10.4 COMPLIANCE WITH TRIAL TREATMENT 48
10.5 ACCOUNTBILITY OF THE TRIAL TREATMENT 48
10.6 CONCOMITANT MEDICATION 48
10.7 EMERGENCY MEDICATION AND PROCEDURES 48
10.8 POST-TRIAL TREATMENT 49
10.9 OTHER TREATMENTS (NON-IMPS) 49
10.10 OTHER INTERVENTIONS 49
11 SAFETY REPORTING 49
11.1 SAFETY REPORTING DEFINITIONS 49
11.2 CAUSALITY ASSESSMENT 50
11.3 SEVERITY ASSESSMENT 51
11.4 PROCEDURES FOR COLLECTING AND RECORDING ADVERSE EVENTS 51
11.4.1 E-DIARY AEs 52
11.4.2 OBSERVATION RELATED AEs 53
11.4.3 VISIT ELICITED AEs 53
11.4.4 LABORATORY AEs 53
11.4.5 NOTES ON RECORDING AEs 53
11.4.6 FOLLOWING UP OF AEs 54
11.5 REPORTING PROCEDURES FOR SERIOUS ADVERSE EVENTS 54
11.6 EXPECTEDNESS 54
11.7 SUSAR REPORTING 54
11.8 FORSEEABLE ADVERSE REACTIONS 55
11.9 DEVELOPMENT SAFETY UPDATE REPORTS 55
11.10 SAFETY PROFILE REVIEW 55
11.11 TRIAL MANAGEMENT GROUP 55

Hanumunthadu B, *et al. BMJ Open* 2023; 13:e072938. doi: 10.1136/bmjopen-2023-072938

11.12 DATA SAFETY MONITORING COMMITTEE (DSMC) 55
11.13 OTHER SAFETY REVIEWS 56
11.14 GROUP HOLDING RULES 56
11.15 INDIVIDUAL HOLDING RULES 57
11.16 STOPPING RULES 58
12 STATISTICS 59
12.1 DESCRIPTIVE STATISTICAL METHODS 59
12.2 THE NUMBER OF PARTICIPANTS 59
12.3 THE LEVEL OF STATISTICAL SIGNIFICANCE 59
12.4 CRITERIA FOR TERMINATION OF TRIAL 59
12.5 PROCEDURE FOR ACCOUNTING FOR MISSING, UNUSED, AND SPURIOUS 59
12.6 INCLUSION IN ANALYSIS 59
12.7 INTERIM ANALYSIS 60
13 DATA MANAGEMENT 60
13.1 DATA INTEGRITY 60
13.2 DATA ARCHIVING AND STORAGE 60
13.3 SOURCE DATA 60
13.4 ACCESS TO DATA 61
13.5 DATA RECORDING AND RECORD KEEPING 61
14 QUALITY ASSURANCE PROCEDURES 62
14.1 RISK ASSESSMENT 62
14.2 MONITORING 62
15 PROTOCOL DEVIATIONS 62
16 SERIOUS BREACHES 62
17 ETHICAL AND REGULATORY CONSIDERATIONS 63
17.1 DECLARATION OF HELSINKI 63
17.2 GUIDELINES FOR GOOD CLINICAL PRACTICE 63
17.3 APPROVALS 63
17.4 TRANSPARENCY IN RESEARCH 63
17.5 REPORTING 63
17.6 PARTICIPANT CONFIDENTIALITY 64
17.7 PARTICIPANT REIMBURSEMENT 64
18 FINANCE AND INSURANCE 64
18.1 FUNDING 64
18.2 INSURANCE 64
18.3 CONTRACTUAL ARRANGEMENTS 64
19 PUBLICATION POLICY 64

20 DEVELOPMENT OF A NEW PRODUCT/ PROCESS OR THE GENERATION OF INTELLECTUAL PROPERTY 65
21 REFERENCES 66

1 KEY TRIAL CONTACTS

**Chief Investigator**	**Dr Maheshi Ramasamy**<br>Consultant Physician, Oxford University Hospitals NHS Foundation Trust<br>Senior Clinical Researcher, Oxford Vaccine Group, University of Oxford, Centre for Clinical Vaccinology and Tropical Medicine (CCVTM), Churchill Hospital, Oxford, OX3 7LE, United Kingdom.<br>maheshi.ramasamy@paediatrics.ox.ac.uk
**Sponsor**	University of Oxford<br>Research Governance, Ethics and Assurance<br>Joint Research Office<br>Boundary Brook House<br>Churchill Drive<br>Headington<br>Oxford OX3 7GB<br>United Kingdom
**Funder(s)**	EU Framework Programme for Research and Innovation, Horizon2020, Vacc-iNTS no 815439
**Clinical Trials Unit**	Oxford Vaccine Group, University of Oxford,<br>Centre for Clinical Vaccinology and Tropical Medicine (CCVTM),<br>Churchill Hospital, Oxford, OX3 7LE, United Kingdom
**Statistician**	**Dr Xinxue Liu**<br>Senior statistician,<br>Oxford Vaccine Group, University of Oxford,<br>Centre for Clinical Vaccinology and Tropical Medicine (CCVTM),<br>Churchill Hospital, Oxford, OX3 7LE, United Kingdom
**DSMC Chair**	**Prof Robert Heyderman**<br>Professor of Infectious Diseases & International Health,<br>Head of the Research Department of Infection<br>NIHR Senior Investigator<br>Division of Infection and Immunity<br>University College London<br>Cruciform Building<br>Gower Street<br>London WC1E 6BT<br>Email: r.heyderman@ucl.ac.uk

Hanumunthadu B, *et al. BMJ Open* 2023; 13:e072938. doi: 10.1136/bmjopen-2023-072938

2 LAY SUMMARY

Nontyphoidal Salmonellae are types of bacteria that can cause gut infections resulting in diarrhoea, both in the UK and globally. However, under some circumstances, these bacteria can cause a more severe illness where infection spreads beyond the gut into the blood stream, a condition termed invasive non-typhoidal Salmonellosis (iNTS). iNTS disease is an under-recognised cause of disease and death in Sub Saharan Africa. In these regions, it primarily occurs in young children, particularly those with malaria and malnutrition. High death rates, difficulties in diagnosing this infection in the developing world, increasing resistance of the bacteria to common antibiotics, and spread via contaminated food and water make development of an effective and affordable vaccine against iNTS an essential control measure.

A new and innovative vaccine (iNTS-GMMA), has been developed which is based on the formation of bacterial outer surface particles. This vaccine facilitates exposure of components of the bacteria to the human immune system without the risk of causing infection. Developed by GSK Biologicals and GSK Vaccines Institute for Global health (GVGH), the aim of this vaccine is to confer immune protection to the most common African strains of the bacteria causing iNTS disease.

This study is a first-in-human clinical trial involving 30-42 healthy adult participants who will be randomly allocated to receive either iNTS-GMMA or a placebo. The main objective of this trial is to evaluate the safety of the iNTS-GMMA vaccine in healthy adults in the UK. The secondary objective is to investigate the human immune response to iNTS-GMMA vaccine.

Hanumunthadu B, *et al. BMJ Open* 2023; 13:e072938. doi: 10.1136/bmjopen-2023-072938

Date and version No: 13January2023 Version 3.1

3 SYNOPSIS

Trial Title	**A Phase 1 Clinical Study to Determine the Safety and Immunogenicity of a Novel GMMA Vaccine Against Invasive Non-Typhoid Salmonella**
Internal ref. no. (or short title)	**OVG2020/01 Sal**monella **V**accine Study in **O**xford (SALVO)
Trial registration	ISRCTN51750695
Sponsor	University of Oxford<br>Research Governance, Ethics and Assurance<br>Joint Research Office<br>Boundary Brook House<br>Churchill Drive<br>Headington<br>Oxford OX3 7GB<br>United Kingdom
Funder	EU Framework Programme for Research and Innovation, Horizon2020, Vacc-iNTS<br>The GSK Vaccines Institute for Global Health (Monitoring Costs Only)
Clinical Phase	Phase I, First in Humans
Trial Design	Single centre, participant-observer blind, randomised placebo-controlled safety and immunogenicity interventional study
Trial Participants	Healthy adults, aged 18-55 years inclusive
Sample Size	• 30-42 participants.<br>• Up to 12 participants randomised 1:1 to receive lower dose at 10.6µg (3.8 dilution of full dose) OAg of iNTS GMMA vaccine or a placebo.<br>• Up to 12 participants randomised 1:1 to receive the full dose at 40µg OAg of iNTS GMMA vaccine or a placebo<br>• Eighteen participants randomised 2:1 to receive either the lower dose or full dose dependant on DSMC review versus placebo.
Planned Trial Period	• Total trial period including data analysis is 36 months.<br>• The intended duration of a participant on the trial is 12 months.<br>• The total duration from first participants first visit to last participants last visit is maximum 18 months.
Primary Objective	To determine the safety and tolerability between two dose levels:<br>• a lower dose of the iNTS-GMMA vaccine (5.3 µg STmGMMA in OAg and 5.3 µg SEnGMMA in OAg, each adsorbed on 0.35mg AL^{3+} / dose in isotonic 20mM Phosphate buffered saline pH 6.5)<br>• a full dose of the iNTS-GMMA vaccine (20 µg STmGMMA in OAg and 20 µg SEnGMMA in OAg, each

	adsorbed on 0.35mg AL^{3+} / dose in isotonic 20mM Phosphate buffered saline pH 6.5);<br>in healthy adults 18-55 years inclusive when given three doses of vaccine at 0, 2- and 6-month intervals.
Secondary Objectives	To investigate the immunogenicity at two dose levels:<br>• a lower dose of the iNTS-GMMA vaccine (5.3 µg STmGMMA in OAg and 5.3 µg SEnGMMA in OAg, each adsorbed on 0.35mg AL^{3+} / dose in isotonic 20mM Phosphate buffered saline pH 6.5)<br>• a full dose of the iNTS-GMMA vaccine (20 µg STmGMMA in OAg and 20 µg SEnGMMA in OAg, each adsorbed on 0.35mg AL^{3+} / dose in isotonic 20mM Phosphate buffered saline pH 6.5);<br>in healthy adults 18-55 years when given three doses of vaccine at 0, 2- and 6-month intervals.
Exploratory Objectives	To further investigate the immunogenicity using exploratory immunological analyses at two dose levels:<br>• a lower dose of the iNTS-GMMA vaccine (5.3 µg STmGMMA in OAg and 5.3 µg SEnGMMA in OAg, each adsorbed on 0.35mg AL^{3+} / dose in isotonic 20mM Phosphate buffered saline pH 6.5)<br>• a full dose of the iNTS-GMMA vaccine (20 µg STmGMMA in OAg and 20 µg SEnGMMA in OAg, each adsorbed on 0.35mg AL^{3+} / dose in isotonic 20mM Phosphate buffered saline pH 6.5);<br>in healthy adults 18-55 years when given three doses of vaccine at 0, 2- and 6-month intervals.
Primary endpoint	The recording and assessment of local and systemic adverse events following administration of each vaccine dose:<br>1. Tenderness and pain at the injection site<br>2. Induration<br>3. Redness<br>4. Swelling<br>5. Headache<br>6. Malaise<br>7. Myalgia<br>8. Nausea and/or vomiting<br>9. Diarrhoea<br>10. Abdominal Pain<br>11. Anorexia<br>12. Arthralgia<br>13. Fatigue<br>14. Fever<br>15. Blood parameters (haematology / biochemistry)<br>16. Any unsolicited symptom(s) not listed above in addition to any other AE, SAE or SUSAR
Secondary endpoints	Immunological assays to study the immune responses to vaccines, including:

	1. Antibody concentration against serovar specific O antigen determined by enzyme linked immunosorbent assay (ELISA) before and after each dose
Exploratory endpoints	Exploratory Immunological assays to study the immune responses to vaccines, including but not limited to:<br>1. Antibody concentration against other potential antigens including porins determined by enzyme linked immunosorbent assay (ELISA) before and after each dose.<br>2. Serum bactericidal antibody (SBA) titre against vaccine homologous strains before and after each dose<br>3. Serum bactericidal antibody (SBA) titre against a panel of other strains before and after each dose<br>4. Functional antibody analyses which may include opsonophagocytic assays and glycosylation before and after each dose<br>5. Quantification of circulating vaccine-induced B-cells responses specific for vaccine antigens before and after each dose<br>6. Quantification of vaccine-induced, antigen specific T-cell responses and associated cytokine production before and after each dose<br>7. Investigate the innate and adaptive response to the iNTS-GMMA vaccine by utilising next generation sequencing of the transcriptome to evaluate the differential gene expression profile and DNA storage for investigation of the genetic associations with the immune response<br>8. Oral fluid antibody concentration against O antigen and porins determined by enzyme linked immunosorbent assay (ELISA) before and after each dose<br>9. Create a human reference serum standard against iNTS for set-up of laboratory antibody assays<br>10. Faecal antibody concentration against O antigen determined by enzyme linked immunosorbent assay (ELISA) in a subset of participants who opt-in to stool sample collection.<br>11. To investigate a potential relationship between the composition of the gut microbiota and vaccination outcome in a subset of participants who opt-in to stool sample collection.
Investigational Medicinal Products	1. iNTS GMMA vaccine<br>2. Comparator (Placebo)
Form of vaccine	Glass vials containing 0.7ml sterile suspension containing either STmGMMA (80 µg/mL in OAg) or SEnGMMA (80 µg/mL in OAg) formulated with Alhydrogel (0.7 mg AL^{3+} / mL) in isotonic 20mM Phosphate buffered saline pH 6.5

Date and version No: 13January2023 Version 3.1

Form of Comparator (Placebo)	Glass vials containing 0.7ml sterile suspension containing Alhydrogel (0.7 mg AL^{3+} / mL) in isotonic 20mM Phosphate buffered saline pH6.5
Dose	Lower Dose: 5.3 µg (OAg) STmGMMA/Alhydrogel + 5.3 µg (OAg) SEnGMMA/Alhydrogel (3.8x dilution of full dose generated by combining 0.5 mL of the two vaccine components into an empty vial and transferring 0.25 mL of the two components into 0.7 mL of the placebo vial, 0.5mL of combined vaccine to be administered)<br><br>Full Dose: 20 µg (OAg) STmGMMA/Alhydrogel + 20 µg (OAg) SEnGMMA/Alhydrogel (generated by combining equal volumes of the two vaccine components in an empty vial, 0.5 mL of combined vaccine to be administered)<br>Comparator (Placebo): 0.5mL to be administered
Route	Intramuscular
Vaccine Schedule	3 doses given at 0, 2 and 6 months

Hanumunthadu B, *et al. BMJ Open* 2023; 13:e072938. doi: 10.1136/bmjopen-2023-072938

4 ABBREVIATIONS

AE	Adverse event
AR	Adverse reaction
CCVTM	Centre for Vaccinology & Tropical Medicine
CI	Chief Investigator
CRA	Clinical Research Associate (Monitor)
CRF	Case Report Form
CT	Clinical Trials
CTA	Clinical Trials Authorisation
DMSC	Data Monitoring and Safety Committee
DSUR	Development Safety Update Report
ELISA	Enzyme Linked Immunosorbent Assay
GCP	Good Clinical Practice
GMMA	Outer membrane exosome from genetically modified Gram negative bacteria used as an antigen delivery system; GMMA is a pun on the Italian word for jewel or bud.
GP	General Practitioner
GVGH	GSK Vaccines Institute for Global health
HRA	Health Research Authority
IB	Investigators Brochure
ICF	Informed Consent Form
ICH	International Conference on Harmonisation
IMP	Investigational Medicinal Product
IMPD	Investigational Medicinal Product Dossier
iNTS	Invasive Non-typhoidal Salmonella
IRB	Independent Review Board
MHRA	Medicines and Healthcare products Regulatory Agency
NHS	National Health Service
OVG	Oxford Vaccine Group
PI	Principal Investigator
PIL	Participant/ Patient Information Leaflet
PBMC	Peripheral Blood Mononuclear Cell
RGEA	Research Governance, Ethics and Assurance

Date and version No: 13January2023 Version 3.1

R&D	NHS Trust R&D Department
REC	Research Ethics Committee
RSI	Reference Safety Information
SAE	Serious Adverse Event
SAR	Serious Adverse Reaction
SDV	Source Data Verification
SEn	*Salmonella* Enteritidis
SMPC	Summary of Medicinal Product Characteristics
SOP	Standard Operating Procedure
STm	*Salmonella* Typhimurium
SUSAR	Suspected Unexpected Serious Adverse Reactions
TMF	Trial Master File
TMG	Trial Management Group
TOPS	The Over volunteering Prevention System (http://www.tops.org.uk)

Hanumunthadu B, *et al. BMJ Open* 2023; 13:e072938. doi: 10.1136/bmjopen-2023-072938

5 BACKGROUND AND RATIONALE

5.1 INVASIVE NON-TYPHOIDAL SALMONELLA DISEASE

Salmonella enterica is a rod-shaped Gram-negative bacterium that is further classified into approximately 2500 serovars, a number of which can cause human infection. Of these, *Salmonella* Typhi and Paratyphi are the causative agents of enteric fever and together are referred to as Typhoidal Salmonella. Non-typhoidal Salmonellae (NTS), such as *S*. Enteritidis and *S*. Typhimurium most commonly cause a self-limiting gastroenteritis that is indistinguishable from that caused by many other enteric pathogens. However, these organisms can also cause an invasive syndrome with bacteraemia, high fevers and metastatic infection which if untreated can lead to septicaemia and death. Invasive non typhoidal *Salmonella* (iNTS) infections are more common in resource poor settings of sub-Saharan Africa, children, the elderly and in the immunosuppressed, including HIV-infected individuals. Unlike *S*. Typhi and *S*. Paratyphi, whose only reservoir is humans, NTS can be acquired from multiple animal reservoirs including domestically farmed animals[1–3]. However, data from sub-saharan Africa appears to suggest that human-to-human transmission remains the primary mode of dissemination[4].

5.2 BURDEN OF DISEASE

The Global Burden of Disease Study estimates 535,000 annual global cases of iNTS; associated with 77,500 deaths in 2017 alone, representing a higher case fatality rate when compared with non-typhoid *Salmonella* gastroenteritis or typhoidal *Salmonella*[5].

In the UK, NTS infection is a frequent cause of foodborne gastroenteritis outbreaks. Public Health England surveillance data showed that NTS was isolated from 8630 patient samples in 2016[6]. Whilst there are no human seroprevalence studies, it is clear that NTS remains endemic; particularly in livestock and poultry, with multiple veterinary programs implementing control methods such as testing and vaccination[7]. Although less common than in other parts of the world, invasive syndromes do also occur in the UK. Between 2004-2015, there were 2484 iNTS blood isolates in England, with neonates, individuals aged over 65 and men more likely to have a bacteraemia[8]. A case series of 82 iNTS blood cultures at a single site in the UK, found bacteraemias without an intestinal focus to be related to underlying immunosuppression in 80% of cases[9].

By far the highest burden of iNTS disease throughout the world remains in sub-Saharan Africa with 78.8% of global cases[5]. However, this is a likely underestimate of the true burden of disease, given the limited availability of diagnostics (blood cultures) in this setting. In addition, the clinical presentation of invasive disease in children is poorly defined, typically presenting as a febrile illness similar to malaria, enteric fever or pneumonia. Nevertheless, iNTS remains one of the most commonly identified causes of bacteraemia in the region[3]. A study in Malawi focused on adult medical admissions found iNTS to be the most common isolate from blood cultures, contributing to 37% of all bacteraemias in a 12 month period, and associated with a 33% case fatality rate[10]. A study in Ghana in a paediatric population found that 23.5% of bacteraemias were associated with *Salmonella* sp. of which 59% were associated with *Salmonella* Enteritidis[11]. Another paediatric single-centre study in Malawi found a case fatality rate of 20% in invasive *Salmonella* disease, of which 94% was associated with iNTS. The highest incidence was in the under three age group with 81% of cases, with a median age of 16 months[12].

The age at which infection occurs show a bimodal distribution in most African studies[2,13]. The majority of infections are in the under 5 age group with 68.3% of cases[13]; with a second peak in 30-40 years age group, which is believed to be associated with the higher incidence of HIV, malaria and malnutrition[2,13]. The Global Burden of Disease estimates a disproportionably high incidence of approximately 233,400 cases, including 31,630 deaths in the under 5 year olds globally in 2017[5].

In a meta-analysis of 22 African studies of all ages, *Salmonella* sp. was the most prevalent isolate in blood cultures at 29.4 %, with iNTS accounting for 58.4%. Of those patients who had additional HIV testing, iNTS was particularly associated with HIV infection (OR 8.2) compared with *Salmonella* Typhi bacteraemia (OR 0.07)[14]. In addition, studies have shown a decline in iNTS to be associated with a reduction in HIV, as a result of the availability of ART, as well as public health measures designed to minimise the impact of malaria and malnutrition[3,15,16]. *Salmonella* Enteritidis (SEn) and *Salmonella* Typhimurium (STm) represent the most common serovars to cause invasive disease both globally and within sub-Saharan Africa. *Salmonella* Typhimurium isolates with the multi-locus sequence type (MLST) ST313 have emerged as a dominant subtype in sub-Saharan Africa. Genomic analysis of these isolates reveals genomic degradation and pseudogene formation, characteristics consistent with host adaptation and restriction by the bacterium. Along with enhanced invasive and virulence factors, the ST313 subtype is associated with multidrug resistance, potentially contributing to higher incidence and mortality of iNTS in sub-Saharan Africa[3,17]. In contrast, UK isolates are usually MLST ST19 and are commonly associated with gastroenteritis.

5.3 IMMUNO-PATHOGENESIS

It remains unclear why some infections result in local self-limiting disease whilst others cause invasive disease. In adults, iNTS is more common in the immunocompromised, including individuals with HIV, malaria, malnutrition, sickle cell disease and other immunodeficiency states, such as chronic granulomatous disease[2,3].

In children under 5 years of age, acquisition of functional antibody is associated with protection from *Salmonella* infection. Thus the highest peak of infection is between 6 months to 2 years of age, with the incidence declining thereafter. This observation correlates with protection from placentally transferred maternal antibody waning by 6 months and the development of detectable functional antibodies from natural exposure by 16 months of age[18,19]. A study in Malawi found detectable functional antibodies peaked at 35 months. In addition, this study found STm-specific CD4 T helper cells peaked at 14 months then declined, suggesting CD4 T helper cells alone were not protective.

Functional antibodies targeting iNTS can effect bacterial killing though activation of the classical pathway of complement and subsequent membrane attack complex assembly or by opsonisation, facilitating phagocytosis and oxidative burst-mediated intracellular killing. Possible targets for functional antibody include the O antigen (OAg) component of outer membrane lipopolysaccharide (LPS) or outer membrane protein antigens (OMP Ag) or flagella antigen (FliC). Human studies suggest that bactericidal activity correlated with the detectable levels of IgG to Lipopolysaccharide (LPS) but not to outer membrane protein (OMP) or flagella proteins. However antibodies against the FliC flagellar protein and membrane-bound porin proteins such as OmpD have been shown to be protective in mouse models[20,21].

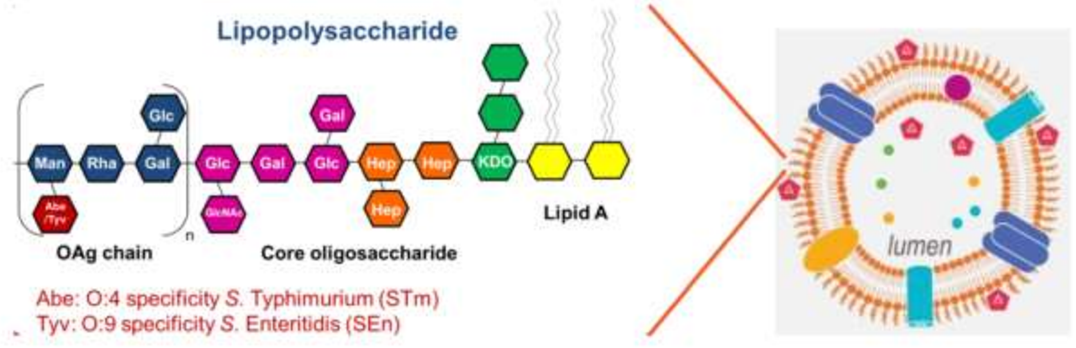

Figure 1.1 Diagram of O Antigen and Lipopolysaccharide constituent of GMMA (taken from Koeberling et al., 2017[22])

Together with lipid A, OAg forms part of the lipopolysaccharide attached to the outer membrane of Salmonella. OAg is the most immunodominant part of LPS and functions as a virulence factor with multiple studies showing attenuation of virulence in bacteria with mutations impairing OAg synthesis and longer OAg chains associated with increased resistance to antibody and complement mediated killing[23]. OAg structure can vary dependant on the individual oligosaccharide units, and this forms the basis of serotyping commonly used in microbiology laboratories. STm is associated with O:4,5 whilst SEn is associated with O:9[24].

5.4 PRIOR EXPERIENCE WITH iNTS VACCINES

The WT05 vaccine produced by Microscience Ltd. is the only iNTS vaccine candidate to date that has progressed to a phase one clinical trial. This dose escalation study randomised participants to receive either an oral attenuated *Salmonella* Typhi or an oral attenuated *Salmonella* Typhimurium at a dose 10^7, 10^8 or 10^9 CFU. Both strains were attenuated with mutations in aroC and ssaV, with ssaV associated with a reduction in function of the type III secretion system. Of the nine participants who received the *Salmonella* Typhimurium oral vaccine only the three participants in the 10^9 group had a significant antibody response[25]. There were no serious adverse events observed and each dose level of oral attenuated Salmonella *Typhimurium* appeared well tolerated. Other vaccines in development include an oral live attenuated vaccine (CVD 1931, CVD 1994), a bivalent OAg conjugate vaccine (COPS, flagellin) and protein vaccines (flagella and OmpD). All remain in the pre-clinical phase[26,27].

5.5 RATIONALE FOR VACCINE BASED ON GMMA TECHNOLOGY

The outer membrane of Gram-negative bacteria such as *Salmonella* naturally release outer membrane vesicles (OMVs) containing outer membrane proteins. OMVs have clear potential as vaccines, displaying immunogenic surface antigens in their natural conformation whilst avoiding the risks of potential infection associated with the use of live attenuated vaccines. Detergent-extracted OMVs from homogenised bacteria have been successfully licensed and used as vaccines against capsular group B meningococcal infections (MenBvac, Bexsero, VA-MENGOC-BC).

In contrast to traditional OMVs, the GMMA-technology derives outer membrane exosomes by induction of hyper blebbing from viable genetically modified bacteria. iNTS GMMA producing

vaccine strains are created from wild-type strains by deletion of *tolR, msbB* and *pagP* genes from *S.* Typhimurium and *S.* Enteritidis. Together, these modifications facilitate increased production of membrane blebs (Δ*tolR*) and reduced-acylation of the lipid A component of bacterial lipopolysaccharide (LPS via Δ*msbB* and Δ*pagP*). GMMA particles with hexa- or penta-acylated LPS induce less cytokine production *in vitro* by human peripheral blood monocytes compared to wild type GMMAs (containing hepta-acyltaled lipid A), which potentially reduces *in vivo* reactogenicity. Furthermore, GMMAs are potentially highly immunogenic, as they present O polysaccharide (OAg) and outer membrane protein antigens identified as immune targets in their native configuration, with minimal expression of cytoplasmic or inner membrane proteins. Purified GMMA particles are filtered, concentrated and implemented as a vaccine[28,29].

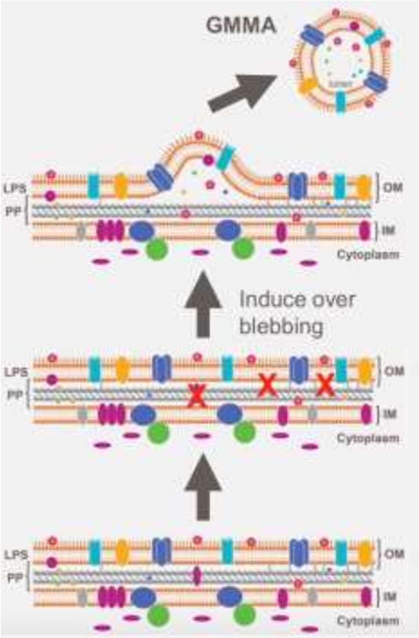

Fig 1.2 Outer membrane blebbing to create GMMA particles (taken from Koeberling 2017[22])

5.6 CLINICAL STUDIES OF ENTERIC GMMA VACCINES

This is the first trial to investigate a 2-component iNTS GMMA vaccine in humans. However, there have been four proof-of-concept clinical trials of a *Shigella* Sonnei GMMA vaccine; three phase 1 trials (including one phase 1 extension trial) in Europe[30,31] and one phase 2a trial in Kenya[32]. Over 100 participants have been enrolled, with all studies demonstrating a well-tolerated vaccine with an acceptable safety profile. The most common adverse event was mild to moderate injection site pain. A transient asymptomatic neutropenia was reported in 9.6% of participants in the two initial phase 1 European trials[30].

In vitro analysis of *Shigella* GMMA have revealed they contain > 95% of the outer membrane and periplasmic proteins[33]. By extrapolation this suggests the iNTS GMMA vaccine may contain a similar percentage of proteins which may contain multiple antigens including OAg and OMP Ag eliciting a potentially broadly protective immune response.

In Phase I trials, the *Shigella* GMMA vaccine used a 3-dose regime with intervals of 0, 1 and 2 months. The iNTS GMMA vaccine will use a 3-dose regimen at 0, 2 and 6 months to allow involution of B cell germinal centres and T cell responses between doses to better investigate

booster and memory responses. The highest *Shigella* GMMA vaccine dose so far tested contained approximately 6 µg OAg and 100 µg protein, while the highest iNTS GMMA dose will contain 40 µg OAg and approximately 31 µg protein.

THE INVESTIGATIONAL PRODUCT: 2-COMPONENT iNTS GMMA SALMONELLA VACCINE

5.7 DESCRIPTION OF INVESTIGATIONAL PRODUCT

The iNTS GMMA Vaccine consists of the outer membrane exosomes of the two most common serovars causing invasive disease, *Salmonella* Enteritidis (O:9) and S*almonella* Typhimurium (O:4.5), adsorbed to Alhydrogel and suspended in isotonic 20mM Phosphate buffered saline pH 6.5. Two doses levels will be used for this study: a full dose of 20 µg STmGMMA as OAg and 20 µg SEnGMMA as OAg on 0.35 mg AL^{3+} / 0.5 ml full dose; and a lower dose at 3.8 dilution rendering a vaccine consisting of 5.3 µg STmGMMA as OAg and 5.3 µg SEnGMMA as OAg on 0.35 mg AL^{3+} / 0.5 ml dose. The placebo matches the vaccine matrix and consists of Alhydrogel (0.35 mg AL^{3+} / 0.5 mL dose) in isotonic 20mM Phosphate buffered saline pH 6.5 without a GMMA component. The vaccine and placebo are both administered as intra-muscular injections.

5.8 CHARACTERISTICS OF VACCINE PREPARATION

STmGMMA and SEnGMMA, the iNTS vaccine active components, are formulated separately at 80 µg GMMA as OAg/ml batches with the GMMA absorbed onto 0.7mg Alhydrogel as Al^{3+}/ml Alhydrogel and suspended in isotonic 20mM Phosphate buffered saline at pH6.5 . Each batch is aliquoted into vials containing 0.7 ml of either STmGMMA/ Alhydrogel or SEnGMMA Alhydrogel, stored at 2-8 °C.

At the clinical site, 0.5ml of SEnGMMA/Alhydrogel and STmGMMA/Alhydrogel will be mixed in an empty vial using a standard operating procedure to yield 1ml of the 2-component iNTS GMMA vaccine containing 40 µg STmGMMA as OAg and 40 µg SEnGMMA as OAg and 0.7 mg Al^{3+} of Alhydrogel suspended in isotonic 20mM Phosphate buffered saline pH 6.5. This procedure including dilution for the lower dose iNT-GMMA vaccine is detailed in the clinical study plan and will be performed at the clinical site by trained study personnel. This procedure has been developed and evaluated by GVGH. Further stability testing has been performed to confirm the quality of the iNTS-GMMA vaccine for up to 6 hours post mixing, however it is envisaged that the final iNTS-GMMA vaccine will be used within 1 hour of mixing.

5.9 IMMUNOGENICITY IN MICE

Anti-OAg ELISA titres correlate with functional antibody assays consistent with immunity in children[18,19]. Mouse studies using challenge with either wild type *Salmonella* Typhimurium or with an OAg knockout strain higher levels of functional bactericidal antibodies in animals exposed to the bacteria expressing OAg [24].

Mice immunised either with STm and SEn GMMA vaccine showed both an elevated OAg titre by ELISA and complement mediated antibody killing via serum bactericidal assay (SBA). These results were then replicated in mice immunised with the two component (STm and SEn) iNTS GMMA vaccine (Figure 1.3) confirming the immunogenicity and validity of the bivalent

Date and version No: 13January2023 Version 3.1

vaccine[34]. On challenge with either live *S.* Typhimurium or *S.* Enteritidis, immunised mice showed a reduction of bacterial burden (CFUs) in the spleen and liver.

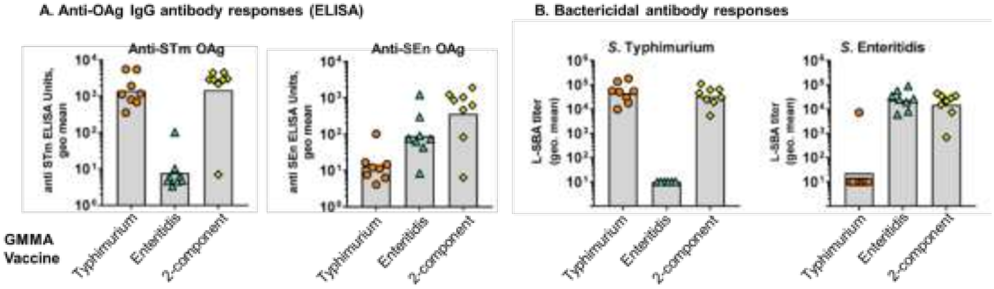

Figure 1.3 (A) Anti-STm and anti-SEn OAg IgG serum antibody responses (ELISA units) and (B) SBA responses against *S.* Typhimurium and *S.* Enteritidis strains. CD1 mice were immunized twice, four weeks apart with 0.16 µg (based on OAg) STmGMMA or SEnGMMA adsorbed on Alhydrogel or a mixture of 0.16 µg each of the two formulated GMMA (2-component iNTS-GMMA). Sera were collected two weeks after the second immunization. Symbols represent results from individual mice, bars represent group geometric mean titres.

In addition there was no significant difference in antibody response when the GMMAs were combined with Alhydrogel[34]

Study	Vaccine	Adsorbent	Dose [µg]	Anti-OAg IgG ELISA titre (geometric mean)
1	STmGMMA	None	2.5	61588
	STmGMMA	Alhydrogel	2.5	69572
2	SEnGMMA	None	2.5	8953
	SEnGMMA	Alhydrogel	2.5	10401

Table 1.1 Anti-OAg IgG ELISA units induced in mice by STmGMMA and SEnGMMA administered with or without Alhydrogel

5.10 IMMUNOGENICITY IN RABBITS

OAg is also a immune target in rabbits. Animals challenged with wild type *Salmonella* Typhimurium or OAg knockout strains develop higher bactericidal antibody response in the wild type arm[24].

In a rabbit immunogenicity study, STm GMMA/Alhydrogel, SEn GMMA/Alhydrogel and the 2-component iNTS-GMMA vaccine were well tolerated and induced strong anti-STm OAg and anti-Sen OAg serum IgG responses and high bactericidal antibody activity against *S.* Typhimurium and *S.* Enteritidis strains.

5.11 TOXICOLOGY

The dosing regimen has been estimated based on immunogenicity in mice and rabbits[24,34], in conjunction with the results of monocyte activation, pyrogenicity and repeat dose toxicology studies in rabbits.

In order to maintain immunogenicity whilst reducing reactogenicity, expression of Lipid A, a potent stimulator of the innate immune system, has been modified via deletions of the genes msbB and pagP. A monocyte activation model with human PBMC stimulated by a mixture of 1:1

STm and SEn iNTS GMMAs produced less IL-6 (a marker of inflammation) than wild-type GMMAs[24]. A similar cytokine release profile was stimulated by unformulated *S. sonnei* 1790-GMMA, which, as Alhydrogel formulated vaccine, were well tolerated in EU and Kenyan adults.

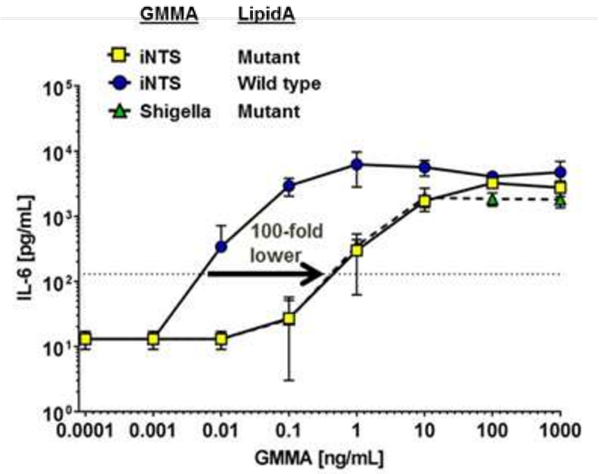

Figure 1.4 Monocyte activation test IL-6 response after incubation with different concentrations of a 1:1 mixture of STmGMMA and SEnGMMA in comparison to *S. sonnei* 1790-GMMA

A rabbit pyrogenicity study was performed using intramuscular administration of undiluted single GMMA formulations and 2-component iNTS-GMMA vaccine. The 2-component iNTS-GMMA vaccine showed a mean peak temperature rise of 1.1^{O}C using the highest target human iNTS dose. This mean peak temperature rise was 0.8 ^{O}C less than the mean peak temperature rise without Alhydrogel, suggesting whilst there is minimal change in immunogenicity, reactogenicity is reduced with the addition of Alhydrogel.

	Group mean initial temperature [°C]	Group mean maximum temperature [°C] with time indicated	Group mean temperature [°C] at end of measurement [5 hours after vaccination]	Mean peak temperature time point after vaccination [minutes]	Mean peak temperature rise [°C]
iNTS-GMMA (STmGMMA/Alhydrogel + SEnGMMA/Alhydrogel)<br>20 µg + 20 µg [OAg]	38.6	39.7	39.6	210	1.1
Unformulated mixture of STmGMMA + SEnGMMA<br>2 µg + 2 µg [OAg]	39.3	41.2	39.8	180	1.9

Date and version No: 13January2023 Version 3.1

Table 1.2 STn+SEn with or without Alhydrogel dosed rabbits, showing a mean temperature rise of 1.1 ^{O}C in STm/Sen with Alhydrogel (iNTS Vaccine)

A repeat dose toxicology study in rabbits has been performed using the highest anticipated human iNTS dose at Covance Laboratories (Harrogate, UK).

Group number	Group description	Dose level (µg GMMA [OAg])	Dose Days	Animal numbers			
				Main necropsy		Recovery necropsy	
1	Control (Saline)	0	1, 15, 29, 43	5 Male	5 Female	4** Male	5 Female
2	Test	20 + 20*	1, 15, 29, 43	5 Male	5 Female	5 Male	5 Female

*The test article was 0.5 mL of a bed-side mixing of SEnGMMA/Alhydrogel and STmGMMA/Alhydrogel each containing 20 µg of each GMMA (quantified based on OAg) and 0.35 mg Al^{3+} Alhydrogel
**One animal was withdrawn during the pre-immunization phase

Main necropsy: Day 46 (3 days after last injection)
Recovery necropsy: Day 71 (28 days after last injection)

Table 1.3 Outline of repeat dose toxicology study in rabbits. INTS GMMA vaccine versus Saline Control Group

New Zealand White rabbits were grouped into a test and control (saline) group. They then received 0.5ml (40µg OAg) of the iNTS-GMMA vaccine at 2 weekly intervals. The results indicated a systemically well-tolerated iNTS GMMA vaccine. Please see the Investigator's Brochure for further details.

5.12 RATIONALE / AIM OF TRIAL

Thirty to forty-two participants in the 18-55 year age group will be recruited into the trial. The trial will follow a dose escalation design with the first group randomised 1:1 to receive a lower dose of 10.6 µg total OAg of iNTS-GMMA vaccine (5.3 µg of STmGMMA as OAg and 5.3 µg of SEnGMMA as OAg) or placebo. A second group will be randomised 1:1 to receive the full intended dose of 40 µg total OAg of iNTS-GMMA (20 µg of STmGMMA as OAg and 20 µg of SEnGMMA as OAg) or placebo. A third group will be randomised 2:1 to receive the iNTS-GMMA vaccine at intended full dose versus a placebo (See 7.Trial Design). An adequate safety review from the Data Safety Monitoring Committee (DMSC) will allow progression of the trial from group 1 to group 2 and from group 2 to group 3. Given there are no current licensed iNTS vaccines, the comparator for this trial will be an Alhydrogel placebo. The last dose of the vaccine will be on Day 168, with a final visit 6 months thereafter. Thus, participants will remain on the trial for 12 months in total.

The aim of the trial is to determine the safety of the iNTS-GMMA vaccine. The immunogenicity of the vaccine will be assessed by OAg ELISA and serum bactericidal assays specific to each vaccine serovar. Additional exploratory immunological assays assessing functional antibody (including but not limited to opsonophagocytosis and glycosylation), B cell and T cell responses will be performed. If the results of this trial indicate a vaccine with an adequate safety profile, progression to a further phase 1 clinical study in an iNTS endemic country will occur.

Hanumunthadu B, et al. BMJ Open 2023; 13:e072938. doi: 10.1136/bmjopen-2023-072938

6 OBJECTIVES AND OUTCOME MEASURES

6.1 PRIMARY OBJECTIVE

To determine the safety and tolerability between two dose levels:
- a lower dose of the iNTS-GMMA vaccine (5.3 µg STmGMMA in OAg and 5.3 µg SEnGMMA in OAg, each adsorbed on 0.35mg AL^{3+} / dose in isotonic 20mM Phosphate buffered saline pH 6.5)
- a full dose of the iNTS-GMMA vaccine (20 µg STmGMMA in OAg and 20 µg SEnGMMA in OAg, each adsorbed on 0.35mg AL^{3+} / dose in isotonic 20mM Phosphate buffered saline pH 6.5);

in healthy adults 18-55 years when given three doses of vaccine at 0, 2- and 6-month intervals.

6.2 SECONDARY OBJECTIVE

To investigate the immunogenicity at two dose levels:
- a lower dose of the iNTS-GMMA vaccine (5.3 µg STmGMMA in OAg and 5.3 µg SEnGMMA in OAg, each adsorbed on 0.35mg AL^{3+} / dose in isotonic 20mM Phosphate buffered saline pH 6.5)
- a full dose of the iNTS-GMMA vaccine (20 µg STmGMMA in OAg and 20 µg SEnGMMA in OAg, each adsorbed on 0.35mg AL^{3+} / dose in isotonic 20mM Phosphate buffered saline pH 6.5);

in healthy adults 18-55 years when given three doses of vaccine at 0, 2- and 6-month intervals.

6.3 EXPLORATORY OBJECTIVES

To further investigate the immunogenicity using exploratory immunological analyses of the two dose levels:
- a lower dose of the iNTS-GMMA vaccine (5.3 µg STmGMMA in OAg and 5.3 µg SEnGMMA in OAg, each adsorbed on 0.35mg AL^{3+} / dose in isotonic 20mM Phosphate buffered saline pH 6.5)
- a full dose of the iNTS-GMMA vaccine (20 µg STmGMMA in OAg and 20 µg SEnGMMA in OAg, each adsorbed on 0.35mg AL^{3+} / dose in isotonic 20mM Phosphate buffered saline pH 6.5);

in healthy adults 18-55 years when given three doses of vaccine at 0, 2- and 6-month intervals

6.4 PRIMARY ENDPOINTS / OUTCOME MEASURES

The recording and assessment of local and systemic adverse events following administration of each vaccine dose;
- Tenderness and pain at the injection site
- Induration
- Redness

- Swelling
- Headache
- Malaise
- Myalgia
- Nausea and/or vomiting
- Diarrhoea
- Abdominal Pain
- Anorexia
- Arthralgia
- Fatigue
- Fever
- Blood parameters (haematology / biochemistry)
- Any unsolicited symptom(s) not listed above in addition to any other AE, SAE or SUSAR

6.5 SECONDARY ENDPOINTS / OUTCOME MEASURES

Immunological assays to study immune responses to vaccines, including:

1. Antibody concentration against serovar specific O antigens determined by enzyme linked immunosorbent assay (ELISA) before and after each dose.

6.6 EXPLORATORY ENDPOINTS / OUTCOME MEASURES

Exploratory Immunological assays to study the immune responses to vaccines, including but not limited to:

1. Antibody concentration against other potential antigens including porins determined by enzyme linked immunosorbent assay (ELISA) before and after each dose .
2. Serum bactericidal antibody (SBA) titres against vaccine homologous strains before and after each dose
3. Serum bactericidal antibody (SBA) titre isogenic strains before and after each dose
4. Functional antibody analyses which may include opsonophagocytic assays and glycosylation before and after each dose
5. Quantification of circulating vaccine-induced B-cells responses specific for vaccine antigens before and after each dose
6. Quantification of vaccine-induced, antigen specific T-cell responses and associated cytokine production before and after each dose
7. Transcriptomic profile analysis after immunization to investigate differential expression of innate, B cell and T cell activation gene modules and DNA storage for investigation of the genetic associations with the immune response
8. Oral fluid antibody concentration against O antigen and porins determined by enzyme linked immunosorbent assay (ELISA) before and after each dose
9. Create a human reference serum standard against iNTS for set-up of laboratory antibody assays

Hanumunthadu B, *et al. BMJ Open* 2023; 13:e072938. doi: 10.1136/bmjopen-2023-072938

Date and version No: 13January2023 Version 3.1

10. Faecal antibody concentration against O antigen determined by by enzyme linked immunosorbent assay (ELISA) in a subset of participants who opt-in to stool sample collection.
11. To investigate a potential relationship between the composition of the gut microbiota and vaccination outcome in a subset of participants who opt-in to stool sample collection.

7 TRIAL DESIGN

7.1 OVERVIEW OF TRIAL DESIGN

This is a first in human, phase 1, single-centre participant-observer blind study to assess the safety and immunogenicity of three administrations of iNTS-GMMA vaccine in healthy adults. Participants will be considered enrolled in the study once their first vaccination has been administered. The total number of participants is 30-42, and individuals will be divided into 3 groups as described below:

1. **Group 1 (PARTICIPANT-OBSERVER BLIND)** - lower dose 10.6 µg total OAg of iNTS-GMMA vaccine (three administrations at D0, D56 and D168). This group will consist of 6 participants subdivided into 3 cohort pairs. Within each pair the two participants will be randomised 1:1 to receive the 10.6 µg total OAg of iNTS-GMMA vaccine or a placebo according to the study plan outlined in section 7.2.1. A favourable DSMC review of the safety data from this arm will be required before commencement of group 2. If the DSMC require further safety data at this dose level a further 6 participants subdivided into 3 cohort pairs may be enrolled.

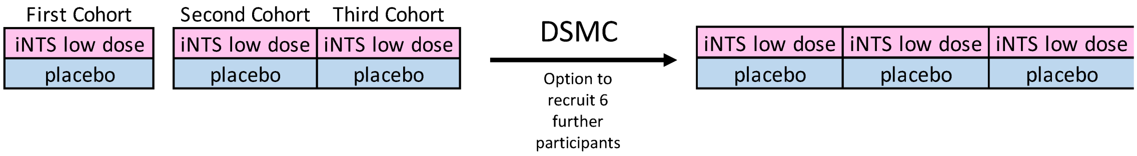

Figure 2.1 Six Participants, subdivided into three cohorts, each cohort consisting of two participants, randomised 1:1 to receive lower dose iNTS-GMMA vaccine or placebo; option to recruit further 6 participants (3 pairs) depending on DSMC review

2. **Group 2 (PARTICIPANT-OBSERVER BLIND)** - full dose 40 µg total OAg of iNTS-GMMA vaccine (three administrations at D0, D56 and D168). This group will only proceed after DSMC review and approval of Group 1. This group will consist of 6 participants subdivided into 3 cohort pairs. Within each pair the two participants will be randomised 1:1 to receive the 40 µg total OAg of iNTS GMMA vaccine or a placebo. See section 7.2.1 for further details regarding safety and dose escalation. A favourable DSMC review of the safety data from this arm will be required before the commencement of group 3. If the DSMC require further safety data at this dose level a further 6 participants subdivided into 3 cohort pairs may be enrolled.

CONFIDENTIAL

Hanumunthadu B, *et al. BMJ Open* 2023; 13:e072938. doi: 10.1136/bmjopen-2023-072938

Date and version No: 13January2023 Version 3.1

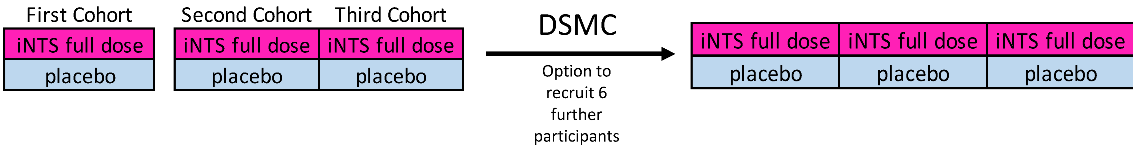

Figure 2.2 Six Participants, subdivided into three cohorts, each cohort consisting of two participants, randomised 1:1 to receive iNTS full dose or placebo; option to recruit further 6 participants (3 pairs) depending on DSMC review

3. **<u>Group 3</u> (PARTICIPANT-OBSERVER BLIND)** – 18 participants will be randomised 2:1 to receive three administrations at D0, D56 and D168 of either iNTS GMMA vaccine (either lower dose or full dose, depending on the safety results of Group 1 and 2) or a placebo. Recruitment to this group will be subject to a favourable interim review of the safety data of group 2 by the DSMC.

   Depending on DSMC review and participant numbers in Group 1 and 2, 18 participants will be enrolled into Group 3:
   Either:

iNTS full dose	iNTS full dose	iNTS full dose	iNTS full dose	iNTS full dose	iNTS full dose
iNTS full dose	iNTS full dose	iNTS full dose	iNTS full dose	iNTS full dose	iNTS full dose
placebo	placebo	placebo	placebo	placebo	placebo

Figure 2.3 Eighteen Participants, randomised 2:1 to receive full dose iNTS-GMMA vaccine or placebo

Or:

iNTS low dose	iNTS low dose	iNTS low dose	iNTS low dose	iNTS low dose	iNTS low dose
iNTS low dose	iNTS low dose	iNTS low dose	iNTS low dose	iNTS low dose	iNTS low dose
placebo	placebo	placebo	placebo	placebo	placebo

Figure 2.4 Eighteen Participants, randomised 2:1 to receive lower dose iNTS-GMMA vaccine or placebo

7.2 DOSE ESCALATION

As this is a first in human trial, we will operate a dose escalation policy in groups 1-3 between lower and full doses. The rationale for these doses is based on experience from animal toxicology studies (5.11 TOXICOLOGY). Escalation between these doses will be dependent on a favourable safety review by the DSMC. This unblinded safety review will consist of AEs from all participants vaccinated in group 1, 2 and 3 at the DSMC review time points indicated below. Group 1 and 2 must have a minimum of 6 participants vaccinated in each group prior to DSMC review. AE Safety Data will include solicited, unsolicited, observation related, laboratory, SAE / SUSARS. Note SAE/SUSARs have specific DSMC reporting instructions as outlined in Section 11.6.

7.2.1 DOSE ESCALATION PROCESS FOLLOWING D0 VACCINE

Group 1 – Lower dose IMP

Initially, 6 participants will be subdivided into 3 pairs. Within each pair the two participants will be randomised 1:1 to receive the 10.6 µg total OAg of iNTS-GMMA vaccine or placebo. Initially

Hanumunthadu B, et al. BMJ Open 2023; 13:e072938. doi: 10.1136/bmjopen-2023-072938

only one pair will be vaccinated and observed for any adverse reactions for 48 hours before further participants are vaccinated. The medically qualified investigator will be asked to provide the decision on whether to proceed after a blinded safety review (on the basis of participant clinical and e-diary reviews) of the first paired cohort. If there are no safety concerns then a second and third pair will be vaccinated at least one hour apart. This will bring the total number of participants in this group to 6, of which 3 receive the lower dose IMP, and 3 receive the placebo.

Group 1: DSMC (Unblinded) Review

A DSMC review will be triggered once at least 7 days of data are available for the first 6 participants in group 1. This review will include the assessment of the profile of adverse events from D0 – D6 and the results of the safety blood tests from D0 (V1), and D7(V3).

Should further safety data be required the unblinded DSMC may request a further 6 individuals in 3 pairs to be vaccinated at the same lower dose, with at least an hour between the vaccinations of each pair. Following a favourable review from the DSMC, enrolment and vaccination to the full dose group 2 will proceed.

If participants in Group 1 develop adverse events which meet the group holding rules as detailed in Section 11.14, the study will be paused, with no further vaccinations administered, pending DSMC review. If a favourable decision is received from the DSMC a substantial amendment would be required to continue further vaccination.

Group 2 – Full dose IMP

In the full dose group, 6 participants will initially be subdivided into 3 pairs as in group 1. Within each pair the two participants will be randomised 1:1 to receive the full dose 40 µg total OAg of iNTS-GMMA vaccine or placebo. Initially only one pair will be vaccinated and observed for any adverse reactions/events for 48 hours before more volunteers are vaccinated. The medically-qualified investigator will be asked to provide the decision on whether to proceed after blinded safety review (on the basis of participant clinical and e-diary reviews) of the first paired cohort. If there are no safety concerns then a further two paired cohorts will be vaccinated with the 40 µg total OAg dose. This will bring the total number of participants in this group to 6, of which 3 receive the full dose IMP, and 3 receive the placebo. The second and third pairs will be vaccinated at least one hour apart.

Group 2: DSMC (Unblinded) Review

A DSMC review will be triggered once at least 7 days of data are available for the first 6 participants in group 2. This review will include the assessment of the profile of adverse events from D0 – D6 and the results of the safety blood tests from D0 (V1), and D7(V3).

Should further safety data be required the unblinded DSMC may request a further 6 individuals in 3 pairs to be vaccinated at the same full dose, with at least an hour between the vaccinations of each pair. Following a favourable review from the DSMC, enrolment and vaccination to the full dose group 3 will proceed.

If a favourable opinion is given for Group 1, but not for Group 2, then the DSMC may allow the ‘lower dose’ to be used in Group 3 instead of the full dose. If favourable decision is received

from the DSMC a substantial amendment would be required to continue further vaccination clearly stating the iNTS-GMMA vaccine dose to be administered.

If participants in Group 2 develop adverse events which meet the group holding rules as detailed in Section 11.14, the study will be paused, with no further vaccinations administered, pending DSMC review. If a favourable decision is received from the DSMC a substantial amendment would be required to continue further vaccination.

Group 3 – Low or Full Dose

In this group, 18 participants will be randomised 2:1 to receive the iNTS GMMA vaccine (either the lower dose or full dose, depending on the safety results of Group 1 and 2) or placebo. The total number of participants in the study will be between 30-42 individuals, depending on the final numbers of participants recruited to group 1 and 2.

7.3 DSMC (Unblinded) REVIEWS

The DSMC will review the unblinded safety data at two further timepoints unrelated to dose escalation decisions: at least seven days after the last participant in group 3 to receive the second vaccine; and at least seven days after the last participant in group 3 to receive the third vaccine.

7.4 GROUP ALLOCATION

Allocation to each group will be decided by order of enrolment into the trial. Participants will be considered enrolled in the trial once they receive their first vaccination. Groups 1, then 2 will be preferentially recruited to ensure study progress as per the dose escalation process above with randomisation within each cohort pair. Once safety data have been reviewed by the DSMC, participants recruited to group 3 will be randomised to receive either iNTS GMMA vaccine (either lower dose or full dose, depending on the safety results of Group 1 and 2) or placebo in a 2:1 ratio.

7.5 SAFETY MONITORING

Safety outcomes of the participants will be monitored throughout the study. This will be done by monitoring symptoms at visits, daily review of an electronic symptom diary up to D6 after every vaccine received on the study, and safety blood tests (Table 2.1).

Group 1 and 2 vaccinations will proceed in a staggered fashion (Fig 2.2), dependent on safety review by the DSMC. The time interval between the 7 days post the last participant to receive their first vaccine administration in Group 1 to the first participant in Group 2 to receive their first vaccine administration therefore allows continuous monitoring of the safety of both the second (D56) and third (D168) vaccinations in Group 1 participants receiving low dose IMP prior to the respective vaccinations in Groups 2.

The medically qualified investigator will be asked to provide the decision on whether to proceed to second (D56) and third (D168) vaccine administration in Group 2 after reviewing 7 days of blinded safety data after the last participant receives the second and third vaccine administrations in Group 1 respectively (See section 11.14). This review will consider blinded safety data from

the results of participant e-diaries, eCRFs, AEs after vaccination and safety bloods. This review will be based on the clinical judgement of the investigator with the option to escalate to the DSMC if any concerns. A similar review will be conducted using the safety data from the last participant to receive their second (D56) and third vaccine (D168) administrations of Group 2 prior to proceeding the the second (D56) and third (D168) vaccine administration of Group 3.

Cumulative toxicity has not been observed with similar GMMA based *Shigella* vaccines and is not expected with this vaccine. Thus staggered vaccination of cohorts of second and third vaccines in Group 1 and 2 will not be performed.

Full details about the reporting of any adverse events or serious adverse events, and the role of the DSMC beyond the dose escalation reviews is discussed in section 11 of the protocol.

Hanumunthadu B, *et al. BMJ Open* 2023; 13:e072938. doi: 10.1136/bmjopen-2023-072938

Date and version No: 13January2023 Version 3.1

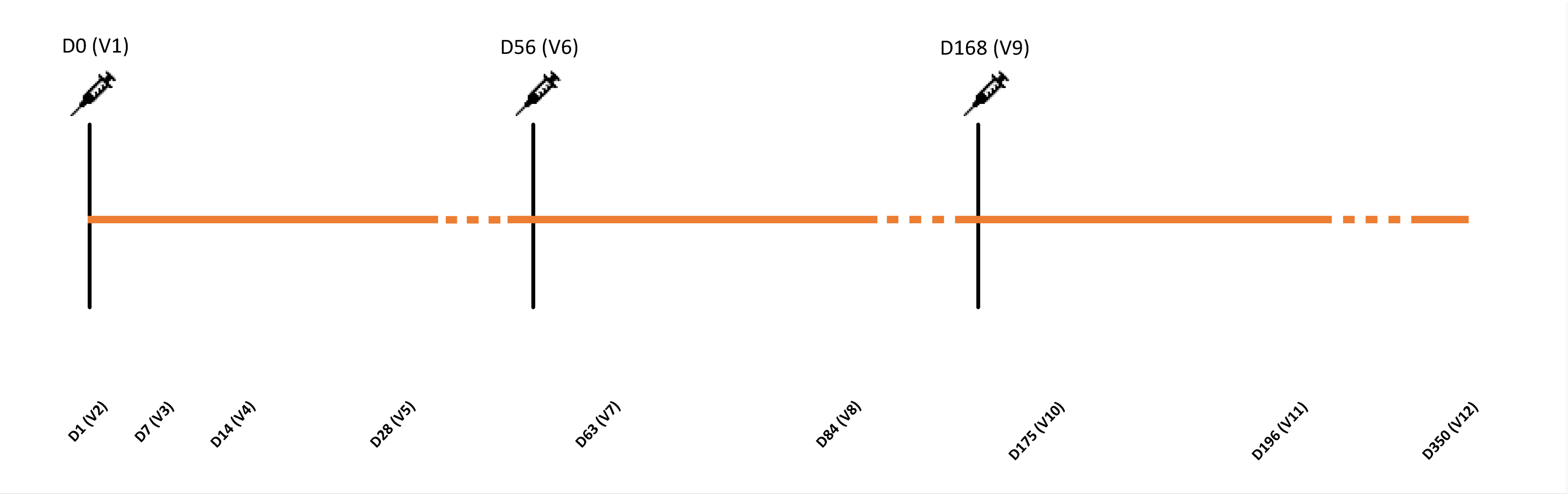

Figure 2.1 Vaccine schedule: Groups 1 – 3

CONFIDENTIAL

Date and version No: 13January2023 Version 3.1

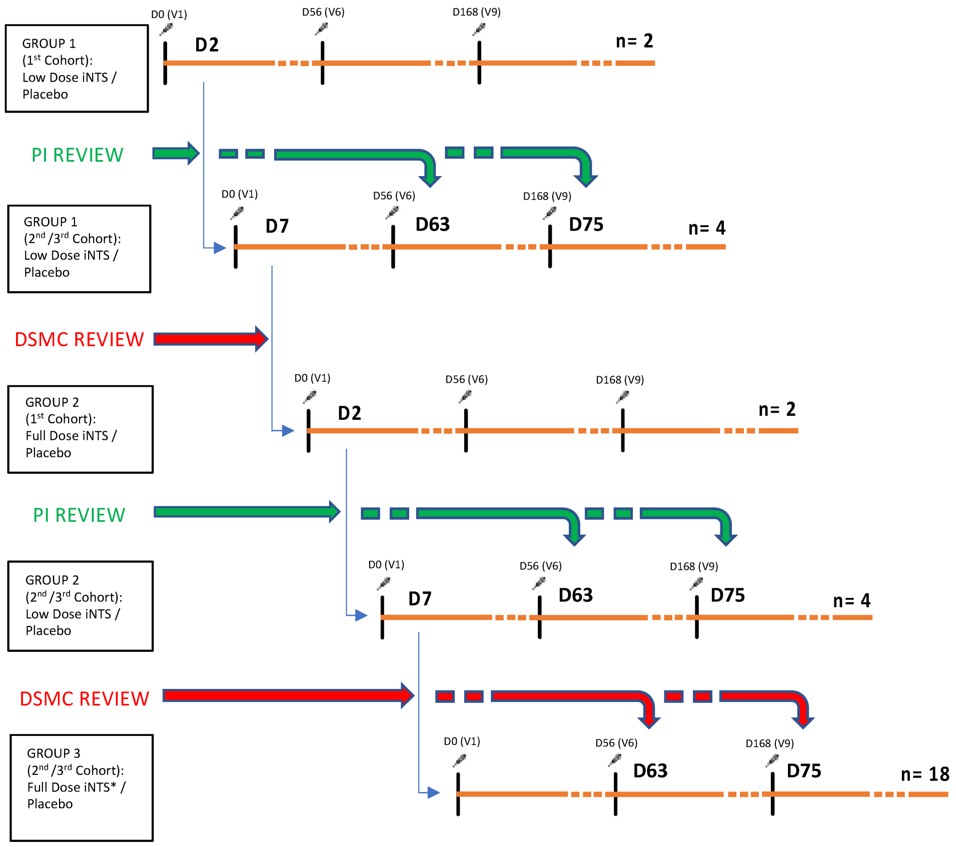

* Low Dose iNTS may be considered if unacceptable safety signal in Group 2 high dose iNTS following Investigator and DSMC review

Figure 2.2 Dose Escalation Schedule: Groups 1 - 3

CONFIDENTIAL

Hanumunthadu B, *et al. BMJ Open* 2023; 13:e072938. doi: 10.1136/bmjopen-2023-072938

	Screening	V1	V2	V3	V4	V5	V6	V7	V8	V9	V10	V11	V12
Indicative Study Day		D0	D1	D7	D14	D28	D56	D63	D84	D168	D175	D196	D350
Day post last vaccine		0	1	7	14	28	56	7	28	112	7	28	182
Visit Window (days)	V1 - 90*		0	+/- 1	+/- 2	+/- 7	+/- 4	+/- 1	+/- 7	+/- 4	+/- 1	+/- 7	+/- 28
Informed consent	x												
Confirmation of eligibilty criteria		x					x			x			
Obtain 24 hr contact details		x					x			x			
Medical history (including demographics and medication)	x												
Interim medical history (including concurrent medication)		x	x	x	x	x	x	x	x	x	x	x	x
Review / collection of AEs and SAEs since last visit		x	x	x	x	x	x	x	x	x	x	x	x
Physical examination	x												
Vital signs	x	x	x	x	x	x	x	x	x	x	x	x	x
Urine pregnancy test	x	x					x			x			
Urine dipstick (with urine analysis as needed)	x												
Blood sample	x	x	x	x	x	x	x	x	x	x	x	x	x
Oral Fluid Swab		x			x	x	x	x	x	x	x	x	x
Stool/Faecal Sample (Optional Only)		x										x	x
**Vaccination**		**x**					**x**			x			
e-Diary entries		x	x	x			x	x		x	x		
Intervention arm allocation		x											

Table 2.1 Visit Structure: Groups 1 - 3

Participants will complete the scheduled visits within the visit windows outlined above, however due to unforeseen circumstances such as participant unavailability, the visit may still proceed outside of window if reasonable to do so, as judged by the investigator. A physical exam is performed at screening. A physical exam is not routinely performed at the remaining visits unless the clinical history or situation deems it necessary.

*Once the screening visit has been completed, V1 must be scheduled within a maximum of 90 days of the date of the screening visit. This interval allows for receipt of GP medical summaries or repeat blood tests. If the V1 for the potential participant falls outside of this period, a repeat screening visit must be performed if the participant remains eligible and willing. However certain tasks which are unnecessary to be repeated as judged by the investigator may be brought forward. Tasks which must be repeated at this re-screening visit include: consent, interim medical history and urine pregnancy test (if applicable). Other tasks may also be performed at the Investigator's discretion.

Hanumunthadu B, *et al. BMJ Open* 2023; 13:e072938. doi: 10.1136/bmjopen-2023-072938

Date and version No: 13January2023 Version 3.1

8 PARTICIPANT IDENTIFICATION

8.1 TRIAL PARTICIPANTS

Male or female participants aged 18-55 years inclusive who are in good health (as determined by a study doctor) and who are able to provide written informed consent, will be eligible for inclusion in this study. Between 30-42 participants are required.

8.2 INCLUSION CRITERIA

Participants must satisfy all of the following criteria to be considered eligible for the study:

- Willing and able to give informed consent for participation in the study
- Aged between 18 and 55 years inclusive
- In good health as determined by
    - Medical history
    - Physical examination
    - Clinical judgment of the investigators
- (Females) Willing to use highly effective contraception as defined in Section 8.5 from one month prior to receiving the first vaccine and for the duration of the study
- Able to attend the scheduled visits and to comply with all study procedures, including internet access for the recording of diary cards
- Willing to allow his or her General Practitioner and/or Consultant, if appropriate, to be notified of participation in the study.
- Willing to allow study team access to medical records for the purposes of eligibility assessment and / or safety follow up during the trial.
- Willing to provide their national insurance number or passport number to be registered on The Over-Volunteering Prevention System (TOPS).

8.3 EXCLUSION CRITERIA

The participant may not enter the study if any of the following apply:

- History of significant organ/system disease that could interfere with the trial conduct or completion in the clinical judgement of the investigators. This includes any history of **significant** disease in the following:
    - Cardiovascular disease including congenital heart disease, previous myocardial infarction, valvular heart disease (or history of rheumatic fever), previous bacterial endocarditis, history of cardiac surgery (including pacemaker insertion), personal or family history of cardiomyopathy or sudden adult death
    - Respiratory disease such as uncontrolled asthma and chronic obstructive pulmonary disease
    - Endocrine disorders such as diabetes mellitus and Addison's disease
    - Significant renal or bladder disease
    - Biliary tract disease

Hanumunthadu B, *et al. BMJ Open* 2023; 13:e072938. doi: 10.1136/bmjopen-2023-072938

  - Gastro-intestinal disease such as inflammatory bowel disease, abdominal surgery within the last two years, coeliac disease and liver disease (including hepatitis B or C infection)
  - Neurological disease such as seizures and myasthenia gravis
  - Haematological disease including coagulation problems
  - Metabolic disease such as glucose-6-phosphate dehydrogenase deficiency
  - Psychiatric illness requiring hospitalisation
  - Depression, anxiety or other psychiatric illness whose severity is deemed clinically significant by the study investigators
  - Known or suspected drug and/or alcohol misuse (alcohol misuse defined as an intake exceeding 42 units per week)
  - Non-benign cancer, except squamous cell or basal cell carcinoma of the skin and cervical carcinoma in situ
- Have any known or suspected impairment or alteration of immune function, resulting from, for example:
  - Congenital or acquired immunodeficiency (including IgA deficiency)
  - Human Immunodeficiency Virus infection or symptoms/signs suggestive of an HIV-associated condition
  - Autoimmune disease
  - Receipt of immunosuppressive therapy such as anti-cancer chemotherapy or radiation therapy within the preceding 12 months or long-term systemic corticosteroid therapy (including for more than 7 days consecutively within the previous 3 months).
- Study significant abnormalities on screening investigations, that are either unlikely to resolve or do not resolve on repeat testing (at the discretion of an Investigator) within the recruitment timeline of the study
- Have received any oral typhoid vaccination (e.g. Ty21a or M01ZH09) within the last 3 years or a paratyphoid vaccine (as part of a clinical trial)
- Have participated in previous typhoid or paratyphoid challenge studies (with ingestion of challenge agent).
- Receipt of a live vaccine within 4 weeks prior to vaccination or a killed vaccine within 7 days prior to vaccination
- Plan to receive any vaccine other than the study vaccine within 4 weeks after any study vaccination (COVID-19 vaccine exempt, see Section 9.14)
- Any history of allergy or anaphylaxis to a previous vaccine or vaccine component
- Receipt of immunoglobulin or any blood product transfusion within 3 months of study start
- Participation in another research study involving an investigational product or that which may compromise the integrity of the study (e.g. significant volumes of blood already taken in previous study) in the past 12 weeks, or are planning to do so within the trial period
- Planned donation of blood/blood products outside of the study and during the trial period.
- Inability, in the opinion of the Investigator, to comply with all study requirements including likelihood of successful venepuncture during the trial
- Female participants who are pregnant, breastfeeding/lactating or planning pregnancy during the course of the study[1]

[1] As defined by CTFG Recommendations related to contraception and pregnancy testing in clinical trials, current document: https://www.hma.eu/fileadmin/dateien/Human_Medicines/01-About_HMA/Working_Groups/CTFG/2020_09_HMA_CTFG_Contraception_guidance_Version_1.1_updated.pdf [accessed 23rd March 2022]

- Weight less than 50kg or a BMI < 18.4 kg/m^2 or a BMI > 40 kg/m^2
- Any other significant disease or disorder which, in the opinion of the Investigator, may:
  - Put the participants at risk because of participation in the study
  - Influence the result of the study
  - Impair the participant's ability to participate in the study

8.4 TEMPORARY EXCLUSION CRITERIA

The following applies to both **initial enrolment** and **subsequent vaccination** visits. If the temporary exclusion resolves within the time constraints of the trial, they can be enrolled and/or progression in the trial can continue.

- Receipt of any systemic corticosteroid (or equivalent) treatment within 14 days prior to vaccination, or for more than 7 days consecutively within the previous 3 months
- Febrile illness (oral temperature ≥37.5°C) or systemically unwell on the day of vaccination
- If a participant is taking systemic antibiotics then the vaccination is postponed until 7 days after the last dose. This does not apply to topical antibiotic preparations
- Use of antipyretics in the 4 hours prior to vaccination
- A laboratory AE considered, in the opinion of the Investigator, requiring of further time and/or investigation to resolve or stabilise prior to a dose of vaccine being administered
- Symptoms of COVID-19, without confirmation of infection (as per current government guidelines) 14 days prior to vaccination visit
- Validated positive (first of episode) SARS-CoV-2 test (NAAT or antigen) within 4 weeks prior to vaccination visit
- Any illness / AE considered, in the opinion of the investigator, requiring of further time and/or investigation to resolve or stabilise prior to a dose of vaccine being administered

8.5 PREGNANCY AND CONTRACEPTION

The possible adverse effects of the iNTS-GMMA vaccine on the outcome of pregnancy are unknown; therefore, pregnant women will be excluded from the study. Women of childbearing potential will be required to use an effective contraceptive measure. Contraception should be maintained during the vaccination period and for the duration of the study. Should a volunteer become pregnant during the trial, she will be followed up for clinical safety assessment with her ongoing consent and in addition will be followed until pregnancy outcome is determined. We would not routinely perform venepuncture in a pregnant volunteer unless there is clinical need.

Male participants with female partners are not required to use barrier methods for the purposes of contraception, as the risks of vaccine excretion are negligible. The active components of the iNTS-GMMA vaccine are the GMMA particles which consist of blebs of the outer membrane of the two Salmonella serovars, S.Enteritidis and S.Typhimurium. As a result, both components lack the necessary machinery for replication in vivo. They have been designed to stimulate an immune response to the antigen(s) contained within the GMMAs. Together with the lack of replicative machinery and a maximum iNTS-GMMA dose of 40µg makes the risk of human teratogenicity/fetotoxicity possible/unlikely.

Hanumunthadu B, *et al. BMJ Open* 2023; 13:e072938. doi: 10.1136/bmjopen-2023-072938

A woman is considered of childbearing potential, i.e fertile, following menarche and until becoming post- menopausal unless permanently sterile. Permanent sterilisation methods include hysterectomy, bilateral salpingectomy and bilateral oophrectomy. A post-menopausal state is defined as no menses for 12 months without an alternative medical cause. A high follicle-stimulating hormone (FSH) level in the post-menopausal range may be used to confirm a post-menopausal state in women not using hormonal contraception or hormonal replacement therapy. However in the absence of 12 months of amenorrhoea, a single FSH measurement is insufficient.

Female volunteers of childbearing potential are required to use a highly effective form of contraception until their last follow-up visit. Acceptable forms of contraception for female volunteers include:

- combined (estrogen and progestogen containing) hormonal contraception associated with inhibition of ovulation (oral/intravaginal/transdermal)
- progestogen-only hormonal contraception associated with inhibition of ovulation (oral/injectable/implantable)
- intrauterine device (IUD)
- intrauterine hormone-releasing system (IUS)
- bilateral tubal occlusion
- vasectomised male partner
- sexual abstinence when this is in line with the preferred and usual lifestyle of the subject. Periodic abstinence (e.g. calendar, ovulation, symptothermal, post-ovulation methods), declaration of abstinence for the duration of exposure to IMP, and withdrawal are not acceptable methods of contraception.

9 TRIAL PROCEDURES

9.1 RECRUITMENT

Identification of study participants
In order to recruit the required cohort of 30-42 participants, several strategies may be employed, including but not limited to:

- Poster advertising: Display of posters advertising the study throughout local hospitals and doctor's surgeries, tertiary education institutions and other public places with the permission of the owner/ proprietor.
- Direct mail-out / SMS/text message / emails: Where mail-outs are used, participants may be identified via the electoral open register, or through National Health Service databases. These include the National Health Applications and Infrastructure Services (NHAIS) via a NHAIS data extract or equivalent. For the NHS databases initial contact to potential participants will not be made by the study team. Instead study invitation material will be sent out on our behalf by an external company, CFH Docmail Ltd (or equivalent company), in order to preserve the confidentiality of potential participants. CFH Docmail Ltd (or equivalent company) is accredited as having exceeded standards under the NHS Digital Data Security and Protection Toolkit (ODS ID – 8HN70). For mail-outs via the electoral register, we will have access to the names and addresses of individuals who are on the open electoral register (only contains the names of registered voters who have not opted out). In this instance, the study team will upload the mailing list to the CFH

Hanumunthadu B, et al. BMJ Open 2023; 13:e072938. doi: 10.1136/bmjopen-2023-072938

Docmail system (or equivalent company), and the study invitation pack will be sent out by CFH Docmail (or equivalent company). Volunteers may also be recruited using direct SMS/text message, or emails to potential participants identified by GPs from their databases.

- Email campaign: We will contact representatives of local tertiary education establishments and local employers and ask them to circulate posters and link to study website by email or hard copy.
- Oxford Vaccine Centre (OVC) database for healthy volunteers: Direct email and link to members of the public who have registered their interest in potentially volunteering for clinical trials conducted by OVC. This secure database is maintained by OVC and members of the public registered here have given consent to have their details recorded and be contacted expressly for this purpose of being notified when a trial opens for recruitment. They understand this is not a commitment to volunteering for any trial they are contacted about.
- Media advertising: Local media, newspaper and website advertisement placed in locations relevant for the target age group with brief details of the study and contact details for further information.
- Website advertising: Description of the study and copy of information booklet on the OVG website.
- Social media: Advertisements placed on OVG or University of Oxford Social media accounts or targeted social media platform advertisements including, but not restricted to, Twitter, Facebook and Instagram
- Exhibitions: Advertising material and/or persons providing information relating to the study will exhibit using stalls or stands at exhibitions and/or fairs, such as University Fresher's Fairs.

Recruitment, approach and initial eligibility assessment of potential study participants
Potential participants who are interested in the study will be able to contact the OVG by telephone, email or trial website for further information. Once an expression of interest has been received by OVG, an information sheet will be sent via mail, email or downloaded from the website by potential participants to read at their leisure. If participants are willing to proceed, they will be initially screened by a website questionnaire and/or telephone before they are invited for a screening and consent visit, where their eligibility will be assessed by member of the clinical research team at the Oxford Vaccine Group. Permission to access the volunteer medical records either via the electronic records system or GP will be sought (if possible) prior to the screening visit. Participants will be asked to sign a secure electronic document (hosted by the REDCap database) which will then be counter-signed by a study team member. Alternatively written permission to access medical records will be sought at the volunteers screening and consent visit.

9.2 INFORMED CONSENT

The participant must personally sign and date the latest approved version of the informed consent form before any study specific procedures are performed. Consent will be sought as described in relevant SOPs.
Written and verbal versions of the participant information booklet and informed consent form will be presented to the participant, detailing no less than:

- the exact nature of and the rationale for performing the study
- implications and constraints of the protocol

- the risks and benefits involved in taking part

It will be clearly stated that the participant is free to withdraw from the study at any time, for any reason and that they are under no obligation to give the reason for withdrawal. The participant will be allowed at least 24 hours to consider the information from when they receive it, and the opportunity to question the researcher, their GP or other independent parties to decide whether they will participate in the study.

The participant will have the opportunity to discuss the study with a medically qualified investigator. Written informed consent will be obtained by means of a dated signature of the participant and a signature of the appropriately trained and delegated clinician. A copy of the signed informed consent will be given to the participant and the original signed form will be retained at the study site.

Participants will be informed that they would also be eligible for BioBank ('Oxford Vaccine Centre Biobank' Southampton & South West Hampshire LREC (B) 10/H0504/25). BioBank is a separate study and optional to all participants of studies conducted by OVC. Separate consent is sought for this.

9.3 BASELINE ASSESSMENTS AT SCREENING

Once informed written consent is obtained, the following baseline assessments and information is collected as part of the assessment of inclusion/exclusion criteria:

- Participant demographics; age, sex and ethnicity
- Travel history (record travel to any country outside of the UK for longer than 2 months; record any travel to Sub-Saharan Africa or South East Asia)
- Medical history
- Contraception; female participants are asked if they are willing to use effective contraceptive measures one month prior to vaccination and for the remainder of the study
- Use of concomitant medication (including over the counter medications, vitamins, illicit drug use and herbal supplements)
- Recording of resting pulse, blood pressure, temperature, weight and height
- Physical examination; cardiovascular, respiratory, abdominal and gross neurological examination
- Urine dipstick (and laboratory analysis if appropriate) and urine pregnancy test
- Blood samples for: haemoglobin count, white cell indices, platelet count, serum sodium, serum potassium, serum urea, serum creatinine, liver function tests, C-reactive protein, HIV, Hepatitis B and C.
- Collect emergency contact details

The medical, vaccination and prescribed medication history are initially based on participant recall. However, with prior participant approval, patient medical summary, vaccination and prescribed medication history will be formally requested from the GP or accessed via the electronic patient record (if available) at the screening visit if not already requested or accessed in advance. In addition, all participant GPs will be notified of their participation in the study.

Consent will be taken to register the participant on The Over-volunteering Prevention System (TOPS) database to guard against the potential for harm that can result from excessive volunteering in clinical trials involving IMPs and blood donations. This will be done using the

participant's National Insurance number or Passport Number. The TOPS database will be checked for any conflicts at screening, however formal registration will be done at enrolment.

9.4 RANDOMISATION

Groups 1 and 2 will be cohorted in pairs randomised 1:1 to receive the lower dose or full dose iNTS GMMA vaccine respectively versus placebo. Once the study has progressed to Group 3, 18 participants will be randomised 2:1 to receive the investigational product versus placebo.

Randomisation will be conducted using an electronic system within the RedCAP database. Participants in Group 1 and 2 will be randomised 1:1 in blocks of two, to meet the total numbers required for each group. Participants in Group 3 will be randomised 2:1 in blocks of three, to meet the total numbers required.

9.5 BLINDING AND CODE-BREAKING

This study will be conducted in an observer and participant blind fashion. The study blind will be maintained from the time of participant randomisation until participant unblinding which will occur once the last participant has completed their final visit.

There will be dedicated blinded and unblinded study teams. Blinded staff will include clinical study doctors (including the CI), study nurses, administrative and laboratory staff who will be directly managing participants and participant samples. Vaccine and placebo will be reconstituted, checked and administered by a dedicated unblinded study team, such that the participant will not be aware of which vaccine they have received. Assays requiring blinding in the laboratory (eg Elispots), will be measured by at least one individual blinded to vaccine or placebo allocation.

Participants and their General Practioners will receive written notification by letter or email of whether they have received the vaccine or placebo at the time of full study unblinding.

Unblinding may also occur at an earlier time point in the event of the occurrence of SAEs, SARs or SUSARs (please see section 11).

In the case of medical emergency the investigator will have direct access to unblinding of the participant(s) by opening of the participant(s) sealed envelope containing their vaccine record. This will be confirmed by electronic unblinding via the REDCap database, which can be performed in the first instance to avoid any delay in unblinding due to a medical emergency or out of hours.

This will be conducted under the guidance of the Data Safety and Monitoring Committee. Unblinding procedures will be conducted in accordance with local OVG SOPs.

9.6 VACCINATION VISITS

Vaccination visits are held at the CCVTM. The visit procedure for the vaccination visits will be as follows:

- Ensure that participant consent remains valid and confirm continued consent.

Hanumunthadu B, *et al. BMJ Open* 2023; 13:e072938. doi: 10.1136/bmjopen-2023-072938

- Measure Weight, Height and Body Mass Index
- Obtain and document interim medical history since the last visit and check eligibility criteria, specifically temporary exclusion to vaccination
- Review for AEs and SAEs since the last visit
- Training on electronic diary card entry and if second or third vaccine review of diary card entries and laboratory AE profile
- Record oral temperature, pulse and blood pressure
- Perform urinary pregnancy test for females
- Perform blood draw
- Perform oral fluid swab
- Optional collection of stool sample +/- supply 'By Post' stool collection kit as required
- Administer vaccine by IM injection into non-dominant deltoid muscle by the second team member in second clinic room (who remains unblinded, following iNTS-GMMA vaccine preparation as per clinical study plan)
- Observe for immediate adverse events for 60 minutes, followed by post vaccine checks including routine observations, review of vaccine site and assessment of wellbeing
- Schedule next visit and re-iterate participant requirements such as return of the Diary Card entries

9.7 NON-VACCINATION VISITS

Other visits may require the following procedures:

- Obtain and document interim medical history since screening and check continued eligibility
- Review for AEs and SAEs since the last visit
- Review eDiary entries and laboratory blood tests
- Record oral temperature, pulse and blood pressure
- Perform blood draw
- Perform oral swab
- Optional collection of stool sample +/- supply 'By Post' stool collection kit as required
- Schedule next visit and re-iterate participant requirements such as eDiary entries

9.8 OUTSIDE OF CCVTM VISITS

Participants will be asked to maintain a diary card describing all (solicited and unsolicited) adverse events up to seven days post vaccination. If there is an ongoing adverse event recorded in the diary the participant will be asked to continue with ediary entries until resolution depending on the nature of the adverse event and feasibility to do so as judged by the clinical investigator. Laboratory results are also entered into a safety results database in real-time or on demand for active monitoring throughout the study by a member of the study team. Each participant will be able to access a member of the study team 24-hours per day via a study-specific emergency number should they have any concerns or are in need of advice.

9.9 LABORATORY INVESTIGATIONS

In addition to blood samples needed for the safe conduct of the trial and assessment of the primary endpoint, blood/oral fluid/stool samples from the participants will also be subjected to laboratory analyses in order to assess the objectives defined in the secondary and exploratory endpoints. The plan for analysis is outlined below, and will be further detailed in a specific analysis plan:

9.9.1 ANALYSIS OF BACTERICIDAL ACTIVITY (GVGH)

The ability of the antibodies in participants serum samples to mediate killing of *S.* Typhimurium and *S.* Enteritidis in the presence of complement (serum bactericidal activity (SBA), will be quantified. The target strains in the SBA assay will be one wild-type strain per serotype in order to elucidate the antigen-specific SBA using a high-throughput luminescence assay developed by GVGH.

9.9.2 FURTHER ANALYSIS OF FUNCTIONAL ANTIBODIES (OVG and Collaborators)

The ability of the antibodies in participants' serum samples to mediate killing of a panel of *Salmonella* bacteria to assess cross protection of the iNTS-GMMA Vaccine. Further exploratory antibody analyses including but not limited to SBAs, opsonophagocytic assay and Fc glycosylation may be performed by OVG and collaborators.

9.9.3 ANALYSIS OF ANTIBODY CONCENTRATIONS AGAINST O-ANTIGENS (GVGH)

Serum IgG antibody responses against OAg from *S.* Typhimurium and *S.* Enteritidis in samples from all subjects at each time point will be analysed by ELISA. Test samples will be analysed at three dilutions and colour change compared with a standard curve made with calibrated human serum pool, included on each assay plate. Anti-OAg responses will be expressed in ELISA units. Plate coating antigens are well characterized OAg purified by GVGH.

9.9.4 ANALYSIS OF ANTIBODY CONCENTRATIONS AGAINST PORIN AND OTHER ANTIGENS (OVG and Collaborators)

Antibody responses against other antigens from *S.* Typhimurium and *S.* Enteritidis in samples from all subjects at each time point may be analysed by ELISA developed and performed by OVG and collaborators. Test samples may be analysed at multiple dilutions and colour change compared with a standard curve made with calibrated human serum pool, included on each assay plate. Antigen responses will be expressed in ELISA Units.

9.9.5 CELLULAR RESPONSES AND CYTOKINE RELEASE (OVG and Collaborators)

Laboratory analyses to quantify the B-cell and T-cell responses specific to STmGMMA and SEnGMMA components of the vaccine will be performed when feasible using peripheral blood mononuclear cells (PBMCs) derived from study participants sampled before, and at several time points after each dose, using the assays described below.

9.9.6 ANALYSES OF B CELL RESPONSES (OVG and Collaborators)

The ability of the STmGMMA, SEnGMMA and the iNTS-GMMA vaccine to stimulate a detectable increase in antigen-specific memory B cells and plasma cells will be enumerated by ELISPOT using plates coated with vaccine antigens (such as individual GMMA or their

respective purified O antigen) or B cell mitogens. The phenotype and kinetics of the B-cell subsets involved in the response will be determined using fluorescent-labelled antibodies in a flow cytometric assay. In addition, other assays to monitor the B-cell immune response to the vaccines may be performed if sufficient samples are available.

9.9.7 ANALYSES OF T CELL RESPONSE AND CYTOKINE RELEASE (OVG and Collaborators)

In order to evaluate the ability of the iNTS-GMMA vaccine to stimulate T cell responses, we aim to quantify when possible vaccine-induced responding T-cells by multicolour flow cytometry and mass cytometry. Effector T cells will be clones and rested for antigen specificity against iNTS bacterial strains and tested for their capacity to recognise proteins contained within individual GMMAs. Moreover, other assays to monitor the T-cell immune response and cytokine release to the vaccines will be performed if sufficient samples are available.

9.9.8 ANALYSIS OF GENE EXPRESSION (OVG and Collaborators)

RNA will be extracted from a small volume of peripheral blood (~1ml) at three study visits (as per Table 3.1) for analysis of gene expression profiles. This analysis will be used to highlight differences in gene expression induced by vaccination and provide insight into the immunobiology of vaccine responses.

9.9.9 ANALYSIS OF GENETIC DETERMINANTS OF VACCINE RESPONSE (OVG and Collaborators)

DNA samples obtained, from peripheral blood, will contribute to a Biobank of samples from multiple different Oxford Vaccine Group studies. These DNA samples will be used to analyse the genetic factors influencing vaccine responses (immunogenicity and reactogenicity). DNA extraction and storage will only occur with the specific consent of participants, and DNA will not be analysed for any other purpose than to assess factors influencing vaccine responses. This specific goal will therefore not contribute to the results of this individual study.

9.9.10 ANALYSIS OF ORAL FLUID ANTIBODY CONCENTRATION AGAINST O ANTIGENS AND PORINS (OVG and Collaborators)

Oral fluid samples will be collected via an oral fluid swab as detailed in the Clinical Study Plan. Antibody responses against OAg and porin Ag from *S.* Typhimurium and *S.* Enteritidis in samples from all subjects at each time point will be analysed by ELISA. OAg and Porin Ag responses will be expressed in Antibody Units.

9.9.11 COLLECTION OF SERUM TO BE USED AS A REFERENCE STANDARD FOR THE SET-UP OF LABORATORY ASSAYS IN CURRENT / FUTURE STUDIES (OVG/GVGH and Collaborators)

Serum samples (30mls) will be collected at Visit 8 (28 days post 2nd Vaccine) from recipients of the iNTS-GMMA vaccine within group 3. Serum will be processed as per OVG SOPs and stored at -80°C prior to transfer to GVGH for the development of the serum standard. Serum IgG responses to *S*. Typhimurium and *S*. Enteritidis will be screened by O Antigen ELISA. Samples

will be selected based on ELISA optical density (OD), characterised, calibrated and pooled. The calibrated human serum pool (or serum standard) will allow development of an ELISA standard curve by which test samples may be compared for quantification of antibody titres.

9.9.12 ANALYSIS OF FAECAL ANTIBODY CONCENTRATION AGAINST O ANTIGENS (OVG and Collaborators)

This will be an optional study procedure for those participants who opt-in to providing a stool/faeces sample and has no bearing on ongoing participation in the study. Stool will be collected using specific containers following the established SOP. Antibody responses against OAg from *S.* Typhimurium and *S.* Enteritidis will be analysed by ELISA. OAg responses will be expressed in Antibody Units.

9.9.13 INVESTIGATION OF IMPACT OF VACCINATION ON GUT MICROBIOTA (OVG and Collaborators)

This will be an optional study procedure for those participants who opt-in to providing said sample and has no bearing on ongoing participation in the study. Stool will be collected using specific containers following the established SOPs for bacterial whole genome sequencing and 16S RNA sequencing.

9.10 SAFETY BLOOD TESTS

All other laboratory tests including FBC, WBC differential counts, C-reactive protein, urea, creatinine, electrolytes, aspartate transaminase (AST), alkaline phosphatase (ALP), alanine transaminase (ALT), bilirubin, will be performed using the OUHFT, NHS laboratories. Blood samples will be collected in assay sample tubes and delivered to OUH clinical laboratories for analysis according to national SOPs.

Samples collected as part of this study may also be used for other exploratory studies of scientific relevance by the OVG laboratory or any of the collaborating laboratories which may include the transfer of samples within and outside the EU. These samples may include oral fluid, serum, extracted DNA and RNA, and PBMCs. Frozen samples will be stored under the ethical approval for this study until study completion. At this time, samples will be transferred to the Oxford Vaccine Centre Biobank subject to participant consent (see Section 9.2). Studies may include further investigation of the inflammatory and immunological response to vaccination.

Date and version No: 13January2023 Version 3.1

9.11 TRIAL PROCEDURE SCHEDULE

Details of which assays are performed on each visit are recorded in the table below. The total volume of blood obtained per patient over the course of the study will be 637.5 – 667.5mls.

Visits	Days	Vaccine / Post Vaccine Days	Antibody Assay: SBA, OAg (GSK)	Antibody / Porin Assays (OVG)	PBMCs (T Cell assays)	PBMCs (B Cell assays)	Functional Antibody Assays / Extracted from PBMCs	Serum Standard*	Transcriptomics	FBC	U&E, LFT, CRP	Viral Serology	Oral OAg/Porin IgG/A Assays	Stool/Faecal Ig Assays/Microbiota	Total (mls)
Screen	-	-	-	-	-	-	-	-	-	1	2	4	-	-	7
V1	D0	Vaccine 1	10	5	50	-	+	-	2.5	1	2	-	+	+	70.5
V2	D1	+1	-	-	-	20	-	-	2.5	-	-	-	-	-	22.5
V3	D7	+7	10	-	24	10	-	-	2.5	1	2	-	-	-	49.5
V4	D14	+14	-	5	24		-	-	-	-	-	-	+	-	29
V5	D28	+28	10	5	24	20	+	-	-	1	2	-	+	-	62
V6	D56	Vaccine 2	10	5	24	20	+	-	-	1	2	-	+	-	62
V7	D63	+7	10	5	24	10	-	-	-	1	2	-	+	-	52
V8	D84	+28	10	5	24	20	+	30*	-	1	2	-	+	-	62 (92*)
V9	D168	Vaccine 3	10	5	24	20	+	-	-	1	2	-	+	-	62
V10	D175	+7	10	5	24	10	-	-	-	1	2	-	+	-	52
V11	D196	+28	10	5	24	10	+	-	-	1	2	-	+	+	58
V12	D350	+182	10	5	24	10	+	-	-	-	-	-	+	+	49
															637.5 (667.5*)

Table 3.1: Blood Sampling (Including Oral Swab) Schedule (in mls)
***Serum standard (30mls) will be collected from participants in Group 3 only**
** Stool Sample collection will be participant opt-in only

Sampling time points, volumes and investigations may vary at the discretion of the PI. Samples may be omitted as per the investigating team's discretion for example if participants develop low haemoglobin as defined and managed as per the SALVO clinical study plan.

9.12 EARLY DISCONTINUATION / WITHDRAWAL OF PARTICIPANTS

Each participant can exercise their right to withdraw from the study at any time. In addition, the investigator may discontinue a participant from the study at any time if the investigator considers it necessary for participant safety including, though not exclusive to, the following:

- Significant non-compliance with study requirements
- Consent withdrawn
- Lost to follow up

Withdrawal from the study will not result in exclusion of the data generated by that participant from analysis. The reason for withdrawal, if given, will be recorded in the CRF. A participant who is withdrawn from the study can be replaced if the individual has not received any vaccine. Therefore, lost to follow up subjects who have received at least one dose of vaccine will not be replaced. Furthermore, in circumstances pertaining to the safety of the participant, the

Hanumunthadu B, *et al. BMJ Open* 2023; 13:e072938. doi: 10.1136/bmjopen-2023-072938

investigator may choose to discontinue further vaccination +/- study procedures, however with ongoing consent may continue to monitor for safety via either scheduled or unscheduled visits. Such circumstances may include though not exclusive to the following:

- Pregnancy
- An adverse event which requires discontinuation of the study vaccinations or results in an inability to continue to comply with study procedures
- Ineligibility (either arising during the study or in the form of new information not declared or detected at screening)

Withdrawal from the study will not result in exclusion of the data generated by that participant from analysis. All data and participant samples obtained up to the point of withdrawal will be used in the analysis.

9.13 DEFINITION OF END OF TRIAL

The end-of-study is completion of the last laboratory assay on the last participant sample obtained at Visit 12. End of study must be achieved no later than 8 months after obtaining the last participant last sample at Visit 12.

9.14 SPECIAL CIRCUMSTANCES: COVID-19

9.14.1 STUDY CONDUCT / RISK ASSESSMENT

It is difficult to predict the time course of the COVID-19 pandemic. At all times the safety and welfare of study participants remains paramount.

The Chief Investigator will perform a risk assessment as necessary with relevant parties (e.g. DSMC, Regulatory Authorities or GVGH) on the basis of the current UK COVID-19 situation, to determine:

1. Appropriateness to initiate vaccinations
2. Appropriateness to continue the trial once started
3. Necessity to extend trial duration

Dependant on the prevailing COVID-19 situation, the conduct of the trial may be modified in line with national policy in effect at the time in the interests of participant safety. Such measures may include but are not limited to:

- Pausing further vaccinations
- Modifying study visits and procedures (as detailed in the SALVO Clinical Study Plan) eg: study visits conducted by phone or video calling where appropriate.

Any deviation not outlined in this protocol required due national policy in effect at the time, will require a non-substantial/substantial amendment unless specifically permitted to do otherwise by the Sponsor, MHRA and REC.

9.14.2 COVID-19 INFECTION CONTROL MEASURES AT VISITS

The CCVTM was one of the sentinel sites used by the University of Oxford to implement COVID-19 infection control policies and act as a model for securing a workplace. Further details of infection control procedures including the safe handling of clinic visits, a COVID-19 secure workplace, maintaining staff safety are included in the clinical study plan and OVG SOPs. These will be regularly updated in line with University of Oxford and NHS/UK Government policies.

9.14.3 PARTICIPANTS UNDER QUARANTINE

Given the evolving epidemiological situation both globally and in the UK, should a participant be under quarantine and unable to attend any of the scheduled visits, a telephone/video consultation may be arranged in order to obtain safety and the visit may be re-scheduled, depending on the timelines.

9.14.4 PARTICIPANTS WITH COVID-19 SYMPTOMS

Participants who become symptomatic during follow-up will be instructed to call the study team who will then advise on how to proceed with clinical testing for COVID-19, through the community testing programme, if necessary, as per the Clinical Study Plan. Participants would be expected to report a transient, flu-like illness within 24hours of vaccination. If this reaction should include a fever, we would expect this to resolve within 48hours. If a fever starts and resolves within 48hours of vaccination it will be attributed to the vaccine. If a fever persists for more than 48hours, or starts more than 48hours after vaccination, it will be considered unlikely to be related to vaccination and the participant may be advised to proceed with clinical testing for COVID-19 outside of the study if appropriate.

Participants who develop COVID-19 symptoms and have a positive SARS-CoV-2 test (appropriately validated NAAT or antigen test) after the first vaccination can only receive a subsequent vaccination after a minimum 4 weeks interval from their first positive test of that episode, provided they have had only a mild illness and have fully recovered. Moderate-Severe illness will be defined as 4 or above as per the WHO Clinical Progression Scale (See Appendix D: WHO Clinical Progression Scale for clinical studies of COVID-19). Those who have had moderate-severe disease will not receive further IMP.

In cases of mild or asymptomatic disease, the decision to proceed with booster vaccinations will be at clinical discretion of the investigators, and each case will be evaluated by a study doctor before proceeding (including physical examination and peripheral oxygen saturation recording [Sp02]). The trial clinician must assess that the participant has fully recovered from their illness. Participants must have no ongoing symptoms that could be attributable to their COVID-19 illness and feel that they have fully recovered and are well. For participants who are asymptomatic and have a positive SARS-CoV-2 test (and who remain asymptomatic), a minimum of 4 weeks from positivity will be required before further vaccinations are administered. They will also undergo a physical examination and peripheral oxygen saturation recording before proceeding with IMP.

All relevant details and any required modification in study visits/procedures as per protocol will be documented in a separate adverse event eCRF. Management of these participants will be as detailed above and in the SALVO Clinical Study Plan. Participant follow up and safety reporting will be as detailed in Section 11 Safety Reporting.

9.14.5 PARTICIPANTS INVITED FOR COVID-19 VACCINATION DURING THE TRIAL

Participants who are yet to receive a COVID-19 vaccine but become eligible for this as per UK policy, would be invited to discuss this with the study team.. If agreed by the participant and if it were possible, we would find a mutually agreeable time to receive a COVID-19 vaccine in concert with study timelines and as per exclusion / inclusion criteria (section 8.3). Participants would not be impeded in taking up an offer of a COVID-19 vaccine if offered through the national rollout. If a rollout offer coincides with a planned trial vaccine, the trial vaccine would be rescheduled to 2 weeks before or after the COVID-19 vaccine (whichever was closest to trial schedules). This is in line with the UK Green Book COVID-19 vaccination recommendations for other vaccines which recommends "at least 7 days" interval. It would also minimise the risk of any cross-attribution of reactogenicity, and minimise impact on immunology investigations. If a participant receives a COVID-19 vaccine during the trial, both the vaccine and date of administration will be requested and recorded in the eCRF.

10 TRIAL INTERVENTIONS

10.1 INVESTIGATIONAL MEDICINAL PRODUCT (IMP) DESCRIPTION

10.1.1 iNTS GMMA VACCINE

The vaccine product iNTS-GMMA consists of 2 components: 80 µg OAg/mL STmGMMA and 80 µg OAg/mL SEnGMMA each acceptically formulated independently on Alhydrogel (0.7 mg AL^{3+} / mL) in isotonic 20mM Phosphate buffered saline pH 6.5. Prior to administration, equal volumes of each component are mixed as specified in the clinical study plan to yield:

- Full dose: 20 µg OAg of STmGMMA/Alhydrogel + 20 µg OAg of SEnGMMA/Alhydrogel in 0.5 mL.
- Lower dose: 5.3 µg OAg of STmGMMA/Alhydrogel + 5.3 µg OAg of SEnGMMA/Alhydrogel in 0.5 mL (3.8 dilution of iNTS-GMMA vaccine full dose diluted with placebo).

10.1.2 PLACEBO

The placebo consists of 0.5 mL Alhydrogel in isotonic 20mM Phosphate buffered saline pH6.5. The placebo is also used for vaccine dilution.

10.2 BLINDING OF IMPS

Group 1-3 will operate a participant-observer blind i.e. both the participant and the observer will be unaware of the group allocation. There will be a trained unblinded team who will prepare and administer the iNTS and placebo vaccine. Both the iNTS and placebo vaccine will appear similar at the point of administration to the participant.

10.3 STORAGE OF IMP

The vaccine product requires storage at 2 to 8oC throughout and vaccines will be transported to the OVG after authorised release for use in the clinical trial by the GSK Qualified Person (QP)

Hanumunthadu B, *et al. BMJ Open* 2023; 13:e072938. doi: 10.1136/bmjopen-2023-072938

and study approval by EC and MHRA. All movements of study medication between GVGH and OVG, will be documented in accordance with relevant SOPs.

The study treatment will be stored at the OVG in temperature monitored refrigerators with an auditable temperature record in accordance with the manufacturer's instructions and relevant SOPs. Study fridges are connected to a monitoring system with 24-hour access to staff that are able to move the product in the event of significant temperature deviation, for example fridge malfunction as per OVG 001: Vaccine Receipt, Storage, Cold Chain Maintenance and Return/Deposal.

10.4 COMPLIANCE WITH TRIAL TREATMENT

The study investigational product and placebo will be administered by trained unblinded study personnel and will be documented according to GCP guidelines and relevant SOPs. Issues related to compliance are therefore the responsibility of study personnel who have received appropriate training.

Access to the randomisation and vaccination eCRF of the study database will be password protected and restricted to the unblinded study team . In the unlikely event that accidental unblinding (i.e for reasons not outlined in this protocol) of any blinded study team member occurs, this would be recorded as a protocol deviation, and the study team member will be quarantined from taking part in further blinded study activities, in so far as the study progress is not compromised.

10.5 ACCOUNTBILITY OF THE TRIAL TREATMENT

iNTS-GMMA vaccines, placebo (also used as diluent) and empty vials will be manufactured, packaged, labelled and supplied by GVGH. All vaccines (vials and boxes) are labelled with a label specifying 'for clinical trial use only' and no less than the following:

- The clinical trial identifier (by reference code)
- The content of each vial
- Batch and serial number
- Chief Investigator
- Research site

The vaccine will be delivered and stored at the CCVTM pending authorised release for use in the clinical trial.

10.6 CONCOMITANT MEDICATION

The use of all concomitant medication prescribed or over-the-counter, will be recorded in the CRF. There is no restriction on the use of concomitant medication but the use of some prescribed medicines, such as immune suppressive agents, may result in the withdrawal of the participant at the discretion of the Investigator, while others, such as antibiotics, may result in a temporary exclusion.

10.7 EMERGENCY MEDICATION AND PROCEDURES

Hanumunthadu B, *et al. BMJ Open* 2023; 13:e072938. doi: 10.1136/bmjopen-2023-072938

Participants are required to wait and be observed for one hour after the administration of each vaccine dose for signs of anaphylaxis. All clinical staff are trained and can provide evidence of competency in the acute management of anaphylaxis reactions including the use of intra-muscular adrenaline. This is detailed in relevant SOPs and adrenaline is available at all times of vaccine administration and subsequent observation.

The nearest Accident and Emergency Department is at the Oxford University Hospitals NHS Foundation Trust, which is within minutes by ambulance transfer.

10.8 POST-TRIAL TREATMENT

Study medication will not be continued beyond the trial period.

10.9 OTHER TREATMENTS (NON-IMPS)

No other treatments other than those specified in the protocol above will be administered to trial participants.

10.10 OTHER INTERVENTIONS

No other interventions other than those specified in the protocol above will be administered to trial participants.

11 SAFETY REPORTING

11.1 SAFETY REPORTING DEFINITIONS

Adverse Event (AE)	Any untoward medical occurrence in a participant to whom a medicinal product has been administered, including occurrences which are not necessarily caused by or related to that product.
Adverse Reaction (AR)	An untoward and unintended response in a participant to an investigational medicinal product which is related to any dose administered to that participant.<br><br>The phrase "response to an investigational medicinal product" means that a causal relationship between a trial medication and an AE is at least a reasonable possibility, i.e. the relationship cannot be ruled out.<br><br>All cases judged by either the reporting medically qualified professional or the Sponsor as having a reasonable suspected causal relationship to the trial medication qualify as adverse reactions.

Hanumunthadu B, *et al. BMJ Open* 2023; 13:e072938. doi: 10.1136/bmjopen-2023-072938

Serious Adverse Event (SAE)	A serious adverse event is any untoward medical occurrence that:<br>• Results in death<br>• Is life-threatening<br>• Requires inpatient hospitalisation or prolongation of existing hospitalisation<br>• Results in persistent or significant disability/incapacity<br>• Consists of a congenital anomaly or birth defect.<br>Other 'important medical events' may also be considered serious if they jeopardise the participant or require an intervention to prevent one of the above consequences.<br>NOTE: The term "life-threatening" in the definition of "serious" refers to an event in which the participant was at risk of death at the time of the event; it does not refer to an event which hypothetically might have caused death if it were more severe.
Serious Adverse Reaction (SAR)	An adverse event that is both serious and, in the opinion of the reporting Investigator, believed with reasonable probability to be due to one of the trial treatments, based on the information provided.
Suspected Unexpected Serious Adverse Reaction (SUSAR)	A serious adverse reaction, the nature and severity of which is not consistent with the information about the medicinal product in question set out:<br>• In the case of a product with a marketing authorisation, in the summary of product characteristics (SmPC) for that product<br>In the case of any other investigational medicinal product, in the investigator's brochure (IB) relating to the trial in question.

11.2 CAUSALITY ASSESSMENT

The relationship of each adverse event to the trial vaccine(s) or study procedures must be determined by a CI-delegated blinded clinician / investigator. The relationship of the adverse event with the study procedures will be categorized as not related, possibly related, probably related or definitely related. The delegated clinician will use clinical judgement to determine the relationship using the following definitions:

<table>
<tr><td>Not related</td><td colspan="2">• No temporal relationship to vaccine administration and
• Alternative aetiology (clinical, environmental or other intervention), and
• Does not follow pattern of recognised response to vaccine administration</td></tr>
<tr><td>Related</td><td>Possible</td><td>• Reasonable temporal relationship to vaccine administration, or
• Event not readily explained by alternative aetiology (clinical, environmental or other interventions), or</td></tr>
</table>

		• Similar pattern of response to that seen to vaccine administration.
	Probable	• Reasonable temporal relationship to vaccine administration, ***and*** • Event not readily produced by alternative aetiology (clinical, environment, or other interventions), ***or*** • Known pattern of response with vaccine administration.
	Definite	• Reasonable temporal relationship to vaccine administration or other study procedure, ***and*** • Event not readily produced by alternative aetiology (clinical, environment, or other interventions), ***and*** • Known pattern of response to vaccine administration.

11.3 SEVERITY ASSESSMENT

To ensure no confusion or misunderstanding of the difference between the terms "serious" and "severe", which are not synonymous, the following note of clarification is provided:

The term "severe" is often used to describe the intensity (severity) of a specific event (as in mild, moderate, or severe myocardial infarction); the event itself, however, may be of relatively minor medical significance (such as severe headache). This is not the same as "serious," which is based on the criteria listed in the definition of an SAE in section 11.1 above. Seriousness (not severity) serves as a guide for defining regulatory reporting obligations.

Severity will be assessed by clinical symptoms, signs, diagnosis, laboratory results and observations as per the appendices A, B and C. Use of the appendices will be detailed in the following sections.

11.4 PROCEDURES FOR COLLECTING AND RECORDING ADVERSE EVENTS

Abnormal clinical findings from medical history, examination or blood tests, will be assessed by a blinded delegated clinician / investigator as to their clinical significance using the severity grading criteria for Adverse Events tables (see Appendix A, B, C).

All AEs that are observed by the investigator or reported by the participant irrespective of their relatedness to the study medication will be recorded from the day of vaccination and until 28 days after each vaccination These will be recorded in either the e-diary for the first 7 days after each vaccine, the laboratory safety database or the eCRF. Outside of this window (i.e. from 28 days after each vaccination and until the point of a subsequent vaccination or until the final visit if vaccination course completed), non-serious AEs will only be recorded if they require medical attention (contact with GP, visit to emergency department). These will be recorded in the eCRF. All AEs will be collected and recorded by the blinded study team.

It will be left to the blinded investigator clinical judgment to decide whether or not an AE is of sufficient severity to require the participant's removal from further vaccination / study. Such

judgement may require the unblinding of the investigator +/- the participant as per study procedures.

A participant may also voluntarily withdraw from the study due to what he or she perceives as an intolerable AE. In such an event, Section 9.10 (Early Discontinuation/Withdrawal of Participants) will apply. All AEs that result in a participant's withdrawal from the study will be, subject to participant consent, followed up where possible until a satisfactory resolution occurs, or until a non-study related causality is assigned. This will involve an end of study assessment at which the requirement for further appropriate care under medical supervision will be determined. If required the participant will be referred to their GP for ongoing medical supervision, until symptoms cease or the condition is deemed resolved or stable.

11.4.1 E-DIARY AEs

Solicited adverse events

Solicited AEs are those listed as foreseeable adverse reactions to either iNTS-GMMA vaccine or placebo in section 11.9 below.

- Solicited adverse events will be recorded by the participant in an electronic diary and graded by the participant alone (appendix A) from the time of each vaccine administration for 7 days post-vaccination (day of vaccination and six subsequent days).

Solicited adverse events will be reviewed daily by the clinical study team. These are participant-entered. If further action is required including face-to-face medical review, and/or prescribed medication this will be recorded by the study team in the eCRF. Causality will be assigned by blinded CI-delegated clinician / investigator. Any solicited AE which meets the definition of a SAE will be managed and reported as per Section 11.6.

Unsolicited adverse events

Unsolicted AEs are those that are NOT listed as foreseeable adverse reactions to either iNTS-GMMA vaccine or placebo in section 11.9 below.

- These may be recorded by the participant in an electronic diary from the time of each vaccine administration for 7 days post vaccination.

Unsolicited adverse events will be reviewed at clinic visits. If clarification of any event is required then the study nurse or doctor will seek this from the participant during a clinical visit or by telephone call. These unsolicited adverse events will be recorded in the AE section of the eCRF. Unsolicited adverse events recorded in the e-diary will be severity graded by the participant. Causality will also be assigned by the CI-delegated clinician / investigator as per section 11.2.

Additionally participants will be asked about the occurrence of AEs during visits and if any are elicited (within the period for which the eDiary is open) that have not already been recorded they will be recorded in the eDiary as above.

Hanumunthadu B, *et al. BMJ Open* 2023; 13:e072938. doi: 10.1136/bmjopen-2023-072938

11.4.2 OBSERVATION RELATED AEs

Physical observations (e.g. temperature, blood pressure) of the patient will be taken at each visit (Section 9.6, 9.7). These will be recorded in the eCRF. If abnormal, a severity grading will be automatically assigned by the blinded study team as per Appendix B.

11.4.3 VISIT ELICITED AEs

Participants will be asked about the occurrence of AEs and if any elicited they will be recorded in the eCRF and graded as per Appendix A and B by a blinded CI-delegated clinician / investigator. Any AEs reported outside of the visits will be recorded in the eCRF, except for AEs elicited during the opening period of the eDiary (see Section 11.4.1).

11.4.4 LABORATORY AEs

During the trial, laboratory results will be entered into a password protected database containing all the trial safety blood results. Severity grading for laboratory AEs is defined in Appendix C. Changes in laboratory values will be recorded as AEs if they are of Grade 2 severity or above. Changes of laboratory values of Grade 1 severity may be recorded as AEs if they are judged to be clinically significant by a blinded CI-delegated clinician / investigator.

If a test is deemed clinically significant, it may be repeated, to ensure it is not a single occurrence. If a test remains clinically significant, the volunteer will be informed and advised with regards appropriate medical care. Laboratory results may be out of normal range for a number of reasons (eg hot weather, delayed transit to processing laboratory). Changes in laboratory values will be recorded as AEs if they fall out of prespecified ranges and judged to be clinically significant by a blinded CI-delegated clinician / investigator, if some action (eg repeat testing for likely clinically significant test result, or reduction in blood volume required during blood draws) or intervention is required. There may be certain circumstances where it may be necessary to unblind the participant. Should this be necessary the same procedures will be followed as for unblinding participants for SAEs.
If abnormal laboratory values are the result of pathology for which there is an overall diagnosis then this diagnosis should be reported as one AE only.
A Grade 4 laboratory AE will be considered a SAE.

11.4.5 NOTES ON RECORDING AEs

Pre-existing medical conditions (present prior to enrolment into the study) are considered “concurrent medical conditions” and should not be recorded as AEs. However, if the participant experiences a worsening or complication of the condition, the worsening or complication should be recorded as an AE. Study staff will ensure that the AE term recorded captures the change in the condition (e.g., “worsening of”).

Each AE should be recorded to represent a single diagnosis. Accompanying signs or symptoms (including abnormal laboratory values) should not be recorded as additional AEs.

Any pregnancy occurring during the clinical study and the outcome of the pregnancy should be recorded and followed up for congenital abnormality or birth defect at which point it would fall within the definition of "serious" and the congenital abnormality of birth defect would be reported as an SAE. Pregnancy notification and follow-up reports on pregnancy outcome will be provided to the DSMC with ongoing consent of the participant.

11.4.6 FOLLOWING UP OF AEs

AEs considered related to the active vaccine or placebo will be followed until resolution, the event is considered stable or until non-study causality is assigned. At the end of the study all other ongoing/open AEs will be assessed by a blinded CI-delegated clinician / investigator, to ensure if not already done so, adequate medical follow-up (if required) has been arranged, eg. referral to participant's general practitioner.

11.5 REPORTING PROCEDURES FOR SERIOUS ADVERSE EVENTS

SAEs will be collected throughout the entire trial period (from first vaccination to D350 or withdrawal).

All SAEs must be recorded on a SAE form (paper or electronic) with causality assessed by the blinded investigator and reported by email to the CI. All SAE will be reported to the DSMC Chair (or nominated designee) within 24 hours of discovery or notification of the event. If the SAE is deemed related, the CI will unblind to confirm whether the participant has received study vaccine or placebo. If the CI deems that this is a SUSAR, this will be reported according to the SUSAR reporting procedures below. In the absence of the CI these tasks may be performed by a Co-Investigator.

Additional information received for a case (follow-up or corrections to the original case) need to be detailed on a new SAE form emailed to the CI and DSMC Chair (or nominated designee).

The chair of the DSMC (or nominated designee) will perform an independent review of SAEs and request any further information required in a manner adherent to the procedures and timelines of the DSMC Charter. Documentation of this review will be kept in the TMF. The DSMC will provide independent real-time safety assessment throughout the study as described below.

11.6 EXPECTEDNESS

Expectedness will be determined according to the reference safety information section of the Investigators' Brochure for iNTS-GMMA vaccine. No IMP related SAEs are expected in this study. All SAEs at least possibly related to iNTS-GMMA vaccine will be considered unexpected and be reported to the MHRA and REC as SUSARs within the regulatory timelines, as in section 11.8.

11.7 SUSAR REPORTING

All SUSARs will be reported to the Sponsor, relevant Research Ethics Committee, GVGH, and to the MHRA. Fatal or life-threatening SUSARs must be reported within 7 days and all other SUSARs within 15 days. Any additional relevant information should be sent within eight days of the report.

The CI or Co-Investigator will also inform all investigators concerned of relevant information about SUSARs that could adversely affect the safety of participants.

11.8 FORSEEABLE ADVERSE REACTIONS

The foreseeable ARs following vaccination with iNTS-GMMA vaccine or placebo include, locally; injection site pain/ tenderness, redness, swelling, induration; and systemically, headache, malaise, fever, nausea, vomiting, abdominal pain, anorexia, myalgia, arthralgia, and fatigue.

11.9 DEVELOPMENT SAFETY UPDATE REPORTS

In addition to the expedited reporting above, the CI or CI-delegated study team member shall submit once a year throughout the clinical trial or on request, a Development Safety Update Report (DSUR) to the;

- MHRA
- Research Ethics Committee
- Sponsor (RGEA)
- GVGH

11.10 SAFETY PROFILE REVIEW

The safety profile will be reviewed on a day to day basis by the investigators using a blinded electronic diary, adverse events CRF and safety bloods to date. Any concerns will be referred to the blinded CI. If the CI remains concerned they may consider unblinding and/or escalation to the unblinded DSMC as required.

11.11 TRIAL MANAGEMENT GROUP

The OVG study investigators will form the trial management group (TMG) and will provide on-going management of the trial.

11.12 DATA SAFETY MONITORING COMMITTEE (DSMC)

The DSMC is independent and will review safety data throughout the study according to the DSMC Charter. Specifically, data review will be done as follows:

1. Formal review of the safety profile after 7 days of safety data has been collected from group 1 before progression to group 2, and review of group 2 after 7 days of safety data has been collected before progression to group 3, as described in section 7.
2. Formal review of the safety profile after two further timepoints unrelated to dose escalation decisions: at least seven days after the last participant in group 3 to receive the

second vaccine; and at least seven days after the last participant in group 3 to receive the third vaccine.
3. Independent review following any SAE deemed to be related to the trial active vaccine or placebo.
4. Unscheduled reviews on request of the study management committee at a demand and frequency determined by the severity of reported adverse events.

From these reviews the DSMC will make recommendations to the study investigators on whether there are any ethical or safety reasons why the trial should not continue. A summary of all blinded and unblinded AEs and SAEs to date will be provided to the DSMC on request.

The outcome of each DSMC review will be communicated directly to the TMG and documentation of all reviews will be kept in the TMF. The CI will inform GVGH of the outcome of the DSMC review.

The Chair of the DSMC will also be contacted for advice where the Chief Investigator feels independent advice or review is required.

11.13 OTHER SAFETY REVIEWS

In addition to formal DSMC review, there will be local blinded safety monitoring reviews. As described in section 7.2 and 7.5, there will be formal local blinded reviews of safety data by the CI or CI-delegated clinician / investigator at:

1. Day 2 following vaccination of the first paired cohort in groups 1 and 2, to decide on progression to vaccination of respective second and third paired cohort.
2. Following 7 days of safety data after the last participant to receive their second vaccine in group 1, to decide on progression to administer second vaccines in group 2.
3. Following 7 days of safety data after the last participant to receive their second vaccine in group 2, to decide on progression to administer second vaccines in group 3.
4. Following 7 days of safety data after the last participant to receive their third vaccine in group 1, to decide on progression to administer third vaccines in group 2.
5. Following 7 days of safety data after the last participant to receive their third vaccine in group 2, to decide on progression to administer third vaccine in group 3.

11.14 GROUP HOLDING RULES

Group holding rules are as follows:

SAE

- All Grade 3 adverse events in any individual which is possibly, probably or definitely related to vaccination (i.e. a AR) will be assessed by the CI-designated clinician / investigator to determine whether the event meets the criteria for a SAE related to vaccination (i.e. a SAR) as per Section 11.1. If this criteria is met the this would trigger the following group holding rule.
- An SAE which occurs in any one individual which is possibly, probably or definitely related to vaccination (i.e. a SAR) would trigger a group holding rule.

Hanumunthadu B, *et al. BMJ Open* 2023; 13:e072938. doi: 10.1136/bmjopen-2023-072938

Solicited/unsolicited/laboratory adverse events

Solicited local adverse events:

- If 2 or more doses of the vaccine or placebo at a given time point (Day 0, Day 56, Day 168) within any group are followed by a Grade 3 solicited local adverse event within 7 days after vaccination (day of vaccination and six subsequent days) and persisting at Grade 3 for >48 hrs

Solicited systemic adverse events:

- If 2 or more doses of the vaccine or placebo at a given time point (Day 0, Day 56, Day 168) within any groups are followed by a Grade 3 solicited systemic adverse event beginning within 7 days after vaccination (day of vaccination and six subsequent days) and persisting at Grade 3 for >48 hrs.

Unsolicited adverse events:

- If 2 or more doses of the vaccine or placebo at a given time point (Day 0, Day 56, Day 168) within any group are followed by a Grade 3 unsolicited adverse event within 7 days after vaccination (day of vaccination and six subsequent day) and persisting at Grade 3 for >48 hrs.

Laboratory adverse event:

- If 2 or more doses of the vaccine or placebo at a given time point (Day 0, Day 56, Day 168) within any group are followed by a Grade 3 laboratory adverse event beginning within 3 days after vaccination and not significantly improving (on clinical judgement), persistent or worsening on repeat testing at a clinically appropriate interval.

If the holding rule has been met and following a safety review by the DSMC it is deemed appropriate to restart dosing or to continue only with the lower dose of vaccine, a request to restart dosing with pertinent data must be submitted to the regulatory authority as a request for a substantial amendment. The DSMC safety review will consider:

- The relationship of the AE or SAE to the vaccine.
- The relationship of the AE or SAE to the vaccine dose, or other possible causes of the event.
- If appropriate, additional screening or laboratory testing for other volunteers to identify those who may develop similar symptoms and alterations to the current Study Information Booklet (SIB) are discussed.
- New, relevant safety information from ongoing research programs on the various components of the vaccine.

The local ethics committee, MHRA, GVGH and the Sponsor will be notified if a holding rule is activated or released.

All vaccinated volunteers will be followed for safety until resolution or stabilisation (if determined to be chronic sequelae) of their AEs.

11.15 INDIVIDUAL HOLDING RULES

In addition to the above stated group holding rule, stopping rules for individual volunteers will apply (i.e. indications to withdraw individuals from further vaccinations):

- **Local reactions:**
  - Injection site ulceration, abscess or necrosis

- **Laboratory AEs:**
  - the volunteer develops a Grade 3 laboratory adverse event considered related within 7 days after vaccination, not significantly improving (on clinical judgement), persistent or worsening on repeat testing.

- **Solicited adverse events:**
  - the volunteer develops a Grade 3 systemic solicited adverse event considered related within 7 days after vaccination (day of vaccination and six subsequent days), persisting continuously at Grade 3 for > 48hrs.

- **Unsolicited adverse events:**
  - the volunteer has a Grade 3 adverse event, considered related to vaccination, persisting continuously at Grade 3 for > 48hrs,
  - the volunteer has a Grade 3 adverse event, considered related to vaccination, which is considered a serious adverse event as assessed by the CI-designated clinician / investigator,
  - the volunteer has a serious adverse event considered related to vaccination, or
  - the volunteer has an acute allergic reaction or anaphylactic shock following the administration of vaccine investigational product.

If a volunteer fulfils any of the temporary exclusion criteria (see section 7) at the scheduled time of a second administration of investigational product, the volunteer will not receive the vaccine at that time. The vaccine may be administered to that volunteer at a later date within the time window specified in the protocol (see Table 1) or they may be withdrawn from the study at the discretion of the Investigator.

All vaccinated volunteers will be followed for safety until the end of their planned participation in the study or until resolution or stabilisation (if determined to be chronic sequelae) of their AEs, providing they consent to this.

11.16 STOPPING RULES

The trial will be discontinued in the event of any of the following:

- New scientific information is published to indicate that subjects in the trial are being exposed to undue risks as a result of administration of the IMP, or as a result of the trial procedures or follow-up schedule.
- Serious concerns about the safety of the IMP arise as a result of one or more vaccine related SAE(s) occurring in the subjects enrolled in this or any other on-going trial of the GMMA vaccine delivery system.
- For any other reason at the discretion of the Chief Investigator or DSMC.

Hanumunthadu B, *et al. BMJ Open* 2023; 13:e072938. doi: 10.1136/bmjopen-2023-072938

Additionally, the DSMC can temporarily pause the trial if time is required to reach a decision regarding stopping the trial e.g. to determine causality for SAE.

12 STATISTICS

12.1 DESCRIPTIVE STATISTICAL METHODS

The analyses for this study will be descriptive in purpose and will not include any hypothesis testing or presentation of p values for group comparisons or power calculation.

12.2 THE NUMBER OF PARTICIPANTS

30-42 participants will be recruited to the study allocated to groups 1-3 as detailed in section 7. Participants will be replaced only if they have not received a dose of vaccine. There has been no formal power calculation to determine this figure as the study is primarily descriptive. The number of participants has therefore been chosen to pragmatically reflect logistical and budgetary constraints.

12.3 THE LEVEL OF STATISTICAL SIGNIFICANCE

There will be no statistical significance testing. All confidence intervals for descriptive analyses will be set at 95%.

12.4 CRITERIA FOR TERMINATION OF TRIAL

The Chief Investigator and Data Safety Monitoring Committee will have the right to terminate the study at any time on grounds of participant safety. If the study is prematurely terminated the investigator will promptly inform the participants and will ensure appropriate therapy and follow-up. If the study is halted, the MHRA, GVGH and relevant Ethics Committee will be notified within 15 days of this occurring.

In the event of the trial being terminated early, follow-up of enrolled participants will still continue as detailed in tables 2.1 for safety reasons, with the exception that further vaccination will not be given and study procedures will be modified to monitor safety only.

12.5 PROCEDURE FOR ACCOUNTING FOR MISSING, UNUSED, AND SPURIOUS

All available data will be used in the analyses and there will be no imputations for missing data. Participants will be analysed according to the group to which they were assigned.

12.6 INCLUSION IN ANALYSIS

All participants with any available data will be included in the analyses.

Hanumunthadu B, *et al. BMJ Open* 2023; 13:e072938. doi: 10.1136/bmjopen-2023-072938

12.7 INTERIM ANALYSIS

An interim analysis of the secondary objective, immunogenicity at two dose levels (lower dose and full dose iNTS-GMMA Vaccine) will be performed once enrolement is complete and once all second vaccine + Day 28 (Day 84; V8) samples have been collected from all groups. An interim analysis of some of the exploratory objectives may also be performed dependant on validation of the exploratory assays.

13 DATA MANAGEMENT

The data management aspects of the study are summarised here with details fully described in the Data Management Plan.

The investigators will populate the content of participants' CRFs, which will be in a paper and/or electronic format using an REDCap database (or an appropriate alternative). This database is stored on a secure University of Oxford server and has restricted access and is password-protected with accountability records. This data includes safety data, laboratory data (both clinical and immunological) and outcome data. All information transcribed to and from the REDCap database is by encrypted (Https) transfer.

Each study participant will have a unique screening number which will be allocated following the taking of informed consent and all names and/or identifying details are not included in any study data electronic file. After enrolment the participants will be identified by a study specific participants number which will be determined by enrolment order and initials as a second identifier. Samples sent to laboratories for processing will be identified by a trial number and participant number only.

13.1 DATA INTEGRITY

Data collection and storage will be inspected throughout the study by internal (performed by the Oxford Vaccine Group) and external (Appledown Clinical Research Ltd) monitoring. The Sponsor may also audit the trial data.

13.2 DATA ARCHIVING AND STORAGE

Study data may be stored electronically on a secure server, and paper notes will be kept in a key-locked filing cabinet at the site. All essential documents will be retained for a minimum of 5 years after the study has finished. Volunteers who complete online screening or telephone screening only (before informed consent) will not have data kept beyond the end of the trial. The need to store study data for longer in relation to licensing of the vaccine will be subject to ongoing review. For effective vaccines that may be licensed, we may store research data securely at the site at least 15 years after the end of the study, subject to adjustments in clinical trials regulations. Participants' bank details will be stored for 7 years in line with the site financial policy. De-identified research data maybe be stored indefinitely.

13.3 SOURCE DATA

Hanumunthadu B, et al. BMJ Open 2023; 13:e072938. doi: 10.1136/bmjopen-2023-072938

Source documents are original documents, data, and records from which participants' CRF data is populated. These include, but are not limited to, hospital records (from which medical history and previous and concurrent medication may be summarised into the CRF), clinical and office charts, laboratory and pharmacy records, diaries, microfiches, radiographs, and correspondence. In this study CRF entries will be considered source data where it is the site of the original recording. All documents will be stored safely under strict confidentiality and with restricted access. On all study-specific documents, other than the signed consent and the participant contact sheet, the participant will be referred to by the study participant number/code only.

13.4 ACCESS TO DATA

Direct access will be granted to authorised representatives from the sponsor/host institution (including Appledown clinical Research Ltd), GSK/GVGH and the regulatory authorities to permit trial-related monitoring, audits and inspections.

13.5 DATA RECORDING AND RECORD KEEPING

The investigators will populate the content of participants' CRFs and all the study data will be recorded directly into an Electronic Data Capture (EDC) system (e.g. REDCap, or similar) or onto a paper source document for later entry into EDC if direct entry is not available. Any additional information that needs recording but is not relevant for the CRF (such as signed consent forms etc.) will be recorded on a separate paper source document. All documents will be stored safely and securely in confidential conditions.

The EDC system (CRF data) uses a relational database (MySQL/ PostgreSQL) via a secure web interface with data checks applied during data entry to ensure data quality. The database includes a complete suite of features which are compliant with GCP, EU and UK regulations and Sponsor security policies, including a full audit trail, user-based privileges, and integration with the institutional LDAP server. The MySQL and PostgreSQL database and the webserver will both be housed on secure servers maintained by Oxford Vaccine Group IT personal and local site IT personal. The servers are in a physically secure location in EU and data are backed up on secure servers operated by the University of Oxford IT Services physically located in EU zone. Backups will be stored in accordance with the IT department schedule of daily, weekly, and monthly retained for one month, three months, and six months, respectively. The IT servers provide a stable, secure, well-maintained, and high capacity data storage environment. REDCap is widely-used, powerful, reliable, well-supported system. Access to the study's database will be restricted to the members of the study team by username and password.

Participant's personally identifiable information will be stored in a separate password protected Access databased saved on a secure University of Oxford server. Only Oxford staff have access to the Access database and are permitted for data entry.

Each study participant will have a unique participant number which will be allocated at the time a screening visit is booked and all names and/or identifying details are not included in any study data electronic file. After enrolment the participants will be identified by a study specific participants number and/or code. Samples sent to laboratories for processing will be identified by trial number and participant number only.

Hanumunthadu B, *et al. BMJ Open* 2023; 13:e072938. doi: 10.1136/bmjopen-2023-072938

The study team will use names and contact details to contact participants about the research study, and make sure that relevant information about the study is recorded for their care, in relation to their health during the study and to oversee the quality of the study. At the completion of the study, unless participants consent otherwise (e.g. requesting to be informed of other trials), participant's personal details will not be used to contact them other than exceptional circumstances concerning their safety. If consent is provided by participants to take part in another study carried out by the study site, personal information and medical information including blood test results may be accessed to avoid unnecessary repetition. If participants provide specific consent, we will use personal identifiable data to invite participants for future research.

Bank details will be stored for 7 years in line with University financial policy.

14 QUALITY ASSURANCE PROCEDURES

14.1 RISK ASSESSMENT

The trial will be conducted in accordance with the current approved protocol, GCP, relevant regulations and Standard Operating Procedures. A risk assessment and monitoring plan will be prepared before the study opens and will be reviewed as necessary over the course of the trial to reflect significant changes to the protocol or outcomes of monitoring activities. Approved and relevant SOPs will be used at all clinical and laboratory sites.

14.2 MONITORING

Regular monitoring will be performed by Appledown Clinical Research Ltd according to the trial specific Monitoring Plan. Data will be evaluated for compliance with the protocol and accuracy in relation to source documents as these are defined in the trial specific Monitoring Plan. Following written standard operating procedures, the monitors will verify that the clinical trial is conducted and data are generated, documented and reported in compliance with the protocol, GCP and the applicable regulatory requirements.

15 PROTOCOL DEVIATIONS

A trial related deviation is a departure from the ethically approved trial protocol or other trial document or process (e.g. consent process or IMP administration) or from Good Clinical Practice (GCP) or any applicable regulatory requirements. Any deviations from the protocol will be documented in a protocol deviation form and filed in the trial master file as per SOP.

16 SERIOUS BREACHES

The Medicines for Human Use (Clinical Trials) Regulations contain a requirement for the notification of "serious breaches" to the MHRA within 7 days of the Sponsor becoming aware of the breach.
A serious breach is defined as "A breach of GCP or the trial protocol which is likely to affect to a significant degree –

(a) the safety or physical or mental integrity of the subjects of the trial; or
(b) the scientific value of the trial".

In the event that a serious breach is suspected the Sponsor must be contacted within 1 working day. In collaboration with the CI the serious breach will be reviewed by the Sponsor and, if appropriate, the Sponsor will report it to the REC committee, Regulatory authority, the relevant NHS host organisation and GSK/GVGH within seven calendar days.

17 ETHICAL AND REGULATORY CONSIDERATIONS

17.1 DECLARATION OF HELSINKI

The Investigator will ensure that this trial is conducted in accordance with the principles of the Declaration of Helsinki.

17.2 GUIDELINES FOR GOOD CLINICAL PRACTICE

The Investigator will ensure that this trial is conducted in accordance with relevant regulations and with Good Clinical Practice.

17.3 APPROVALS

Following sponsor approval the protocol, informed consent form, participant information sheet and required material will be submitted to an appropriate Research Ethics Committee (REC), MHRA, regulatory authorities, and host institution(s) for written approval.
The Investigator will submit and, where necessary, obtain approval from the above parties for all substantial amendments to the original approved documents.

17.4 TRANSPARENCY IN RESEARCH

Prior to the recruitment of the first participant, the trial will have been registered on a publicly accessible database.

Results will be uploaded to the European Clinical Trial (EudraCT) Database within 12 months of the end of trial declaration by the CI or their delegate.

Where the trial has been registered on multiple public platforms, the trial information will be kept up to date during the trial, and the CI or their delegate will upload results to all those public registries within 12 months of the end of the trial declaration.

17.5 REPORTING

The CI shall submit once a year throughout the clinical trial, or on request, an Annual Progress Report to the REC, HRA (where required), host organisation, funder (where required) and Sponsor. In addition, an End of Trial notification and summary report will be submitted to the MHRA, the REC, host organisation and Sponsor.

17.6 PARTICIPANT CONFIDENTIALITY

The trial staff will ensure that the participants' anonymity is maintained. The participants will be identified only by a participant ID number and initials on all trial documents and any electronic database. All documents will be stored securely and only accessible by trial staff and authorised personnel. The trial will comply with UK General Data Protection Regulation (GDPR) and Data Protection Act 2018, which requires data to be anonymised as soon as it is practical to do so.

17.7 PARTICIPANT REIMBURSEMENT

Each participant is compensated for their time and for the inconvenience based on the following figures:

- Travel expenses: £15 per visit
- Inconvenience of blood tests: £10 per visit
- Time required for visits: £20 per visit

Remuneration is on a *pro rata* basis should a participant fail to complete all visits and/or study requirements. Each participant can therefore receive a maximum of £585. Payments will be made in instalments after V0, V6, V10, and V12.
Additional reimbursement for unscheduled visits at £45 per visit will be provided. This will not be given unless an unscheduled visit occurs.

18 FINANCE AND INSURANCE

18.1 FUNDING

This clinical trial is funded by a European Union Horizon2020 grant. Additional budget from GVGH will cover necessary costs for monitoring activities not already funded by the Vacc-iNTS European Union Horizon2020 grant.

18.2 INSURANCE

The University has a specialist insurance policy in place which would operate in the event of any participant suffering harm as a result of their involvement in the research (Newline Underwriting Management Ltd, at Lloyd's of London

18.3 CONTRACTUAL ARRANGEMENTS

Appropriate contractual arrangements will be put in place with all third parties.

19 PUBLICATION POLICY

The Investigator will co-ordinate dissemination of data from this study. All publications (e.g., manuscripts, abstracts, oral/slide presentations, book chapters) based on this study will be reviewed by each sub-investigator prior to submission.

20 DEVELOPMENT OF A NEW PRODUCT/ PROCESS OR THE GENERATION OF INTELLECTUAL PROPERTY

Ownership of IP derived from this trial will be in accordance with the Consortium Agreement signed by Beneficiaries of the Horizon2020 Vacc-iNTS EU Grant No 815437.

Date and version No: 13January2023 Version 3.1

21 REFERENCES

1 Balasubramanian Ruchita, Im Justin, Lee Jung-Seok, Jeon Hyon Jin, Mogeni Ondari D., Kim Jerome H., et al. The global burden and epidemiology of invasive non-typhoidal Salmonella infections . *Hum Vaccin Immunother* 2018;**00**(00):1–6. Doi: 10.1080/21645515.2018.1504717.

2 Feasey Nicholas A, Dougan Gordon, Kingsley Robert A, Heyderman Robert S, Gordon Melita A. Invasive non-typhoidal salmonella disease: an emerging and neglected tropical disease in Africa. *Lancet* 2012;**379**(9835):2489–99. Doi: 10.1016/S0140-6736(11)61752-2.

3 Gilchrist James J, MacLennan Calman A. Invasive Nontyphoidal Salmonella Disease in Africa. *EcoSal Plus* 2019;**8**(2). Doi: 10.1128/ecosalplus.ESP-0007-2018.

4 Post Annelies S., Diallo Seydou Nakanabo, Guiraud Issa, Lompo Palpouguini, Tahita Marc Christian, Maltha Jessica, et al. Supporting evidence for a human reservoir of invasive non-Typhoidal Salmonella from household samples in Burkina Faso. *PLoS Negl Trop Dis* 2019;**13**(10):e0007782. Doi: 10.1371/journal.pntd.0007782.

5 Stanaway Jeffrey D., Parisi Andrea, Sarkar Kaushik, Blacker Brigette F., Reiner Robert C., Hay Simon I., et al. The global burden of non-typhoidal salmonella invasive disease: a systematic analysis for the Global Burden of Disease Study 2017. *Lancet Infect Dis* 2019;**19**(12):1312–24. Doi: 10.1016/S1473-3099(19)30418-9.

6 Public Health England. Salmonella data 2007 to 2016. 2018.

7 Mackenzie Grant, Ceesay Serign J., Hill Philip C., Walther Michael, Bojang Kalifa A., Satoguina Judith, et al. A decline in the incidence of invasive non-typhoidal salmonella infection in the gambia temporally associated with a decline in malaria infection. *PLoS One* 2010. Doi: 10.1371/journal.pone.0010568.

8 Katiyo Shannon, Muller-Pebody Berit, Minaji Mehdi, Powell David, Johnson Alan P., De Pinna Elizabeth, et al. Epidemiology and Outcomes of Nontyphoidal Salmonella Bacteremias from England, 2004 to 2015. *J Clin Microbiol* 2018;**57**(1). Doi: 10.1128/JCM.01189-18.

9 Brown M., Eykyn S.J. Non-typhoidal Salmonella Bacteraemia Without Gastroenteritis: a Marker of Underlying Immunosuppression. Review of Casesat St. Thomas' Hospital 1970–1999. *J Infect* 2000;**41**(3):256–9. Doi: 10.1053/jinf.2000.0750.

10 Gordon M.A., Walsh A.L., Chaponda M., Soko D., Mbvwinji M., Molyneux M.E., et al. Bacteraemia and Mortality Among Adult Medical Admissions in Malawi – Predominance of Non-typhi Salmonellae andStreptococcus pneumoniae. *J Infect* 2001;**42**(1):44–9. Doi: 10.1053/jinf.2000.0779.

11 Wilkens Julia, Newman Mercy J., Commey Joseph Oliver, Seifert Harald. Salmonella bloodstream infection in Ghanaian children. *Clin Microbiol Infect* 1997;**3**(6):616–20. Doi: 10.1111/j.1469-0691.1997.tb00467.x.

12 MacLennan Calman A., Msefula Chisomo L., Gondwe Esther N., Gilchrist James J., Pensulo Paul, Mandala Wilson L., et al. Presentation of life-threatening invasive nontyphoidal Salmonella disease in Malawian children: A prospective observational study. *PLoS Negl Trop Dis* 2017. Doi: 10.1371/journal.pntd.0006027.

13 Ao Trong T., Feasey Nicholas A., Gordon Melita A., Keddy Karen H., Angulo Frederick J., Crump John A. Global Burden of Invasive Nontyphoidal Salmonella Disease, 20101. *Emerg Infect Dis* 2015;**21**(6):941–9. Doi: 10.3201/eid2106.140999.

14 Reddy Elizabeth A., Shaw Andrea V., Crump John A. Community-acquired bloodstream infections in Africa: a systematic review and meta-analysis. *Lancet Infect Dis*

CONFIDENTIAL

2010;**10**(6):417–32. Doi: 10.1016/S1473-3099(10)70072-4.

15 Keddy Karen H., Takuva Simbarashe, Musekiwa Alfred, Puren Adrian J., Sooka Arvinda, Karstaedt Alan, et al. An association between decreasing incidence of invasive non-typhoidal salmonellosis and increased use of antiretroviral therapy, Gauteng Province, South Africa, 2003-2013. *PLoS One* 2017. Doi: 10.1371/journal.pone.0173091.

16 Feasey Nicholas A., Everett Dean, Faragher E. Brian, Roca-Feltrer Arantxa, Kang'ombe Arthur, Denis Brigitte, et al. Modelling the contributions of malaria, HIV, malnutrition and rainfall to the decline in paediatric invasive non-typhoidal Salmonella disease in Malawi. *PLoS Negl Trop Dis* 2015. Doi: 10.1371/journal.pntd.0003979.

17 Feasey Nicholas A., Dougan Gordon, Kingsley Robert A., Heyderman Robert S., Gordon Melita A. Invasive non-typhoidal salmonella disease: An emerging and neglected tropical disease in Africa. *Lancet* 2012;**379**(9835):2489–99. Doi: 10.1016/S0140-6736(11)61752-2.

18 MacLennan Calman A., Gondwe Esther N., Msefula Chisomo L., Kingsley Robert A., Thomson Nicholas R., White Sarah A., et al. The neglected role of antibody in protection against bacteremia caused by nontyphoidal strains of Salmonella in African children. *J Clin Invest* 2008;**118**(4):1553–62. Doi: 10.1172/JCI33998.

19 MacLennan Calman A. Antibodies and Protection Against Invasive Salmonella Disease. *Front Immunol* 2014;**5**(December):1–4. Doi: 10.3389/fimmu.2014.00635.

20 Gil-Cruz C., Bobat S., Marshall J. L., Kingsley R. A., Ross E. A., Henderson I. R., et al. The porin OmpD from nontyphoidal Salmonella is a key target for a protective B1b cell antibody response. *Proc Natl Acad Sci* 2009;**106**(24):9803–8. Doi: 10.1073/pnas.0812431106.

21 Goh Yun Shan, L Armour Kathryn. Igg Subclasses Targeting the Flagella of Salmonella Enterica Serovar Typhimurium Can Mediate Phagocytosis and Bacterial Killing. *J Vaccines Vaccin* 2016. Doi: 10.4172/2157-7560.1000322.

22 Koeberling Oliver. Development of a vaccine based on GMMA against invasive nontyphoidal Salmonella disease in sub-Saharan Africa. Available at https://www.coalitionagainsttyphoid.org/wp-content/uploads/2016/07/16-Development-of-a-Vaccine-Based-on-GMMA-Against-Invasive-Non-Typhoidal-Salmonella-Disease-in-Sub-Saharan-Africa.pdf. Accessed August 28, 2019, 2017.

23 Ilg Karin, Endt Kathrin, Misselwitz Benjamin, Stecher Bärbel, Aebi Markus, Hardt Wolf Dietrich. O-antigen-negative Salmonella enterica serovar typhimurium is attenuated in intestinal colonization but elicits colitis in streptomycin-treated mice. *Infect Immun* 2009;**77**(6):2568–75. Doi: 10.1128/IAI.01537-08.

24 Rondini Simona, Lanzilao Luisa, Necchi Francesca, O'Shaughnessy Colette M., Micoli Francesca, Saul Allan, et al. Invasive african salmonella typhimurium induces bactericidal antibodies against O-antigens. *Microb Pathog* 2013;**63**:19–23. Doi: 10.1016/j.micpath.2013.05.014.

25 Hindle Zoë, Chatfield Steven N., Phillimore Jo, Bentley Matthew, Johnson Julie, Cosgrove Catherine A., et al. Characterization of Salmonella enterica derivatives harboring defined aroC and Salmonella pathogenicity island 2 type III secretion system (ssaV) mutations by immunization of healthy volunteers. *Infect Immun* 2002;**70**(7):3457–67. Doi: 10.1128/IAI.70.7.3457-3467.2002.

26 Tennant Sharon M., MacLennan Calman A., Simon Raphael, Martin Laura B., Khan M. Imran. Nontyphoidal salmonella disease: Current status of vaccine research and development. *Vaccine* 2016;**34**(26):2907–10. Doi: 10.1016/j.vaccine.2016.03.072.

27 MacLennan Calman A, Martin Laura B, Micoli Francesca. Vaccines against invasive Salmonella disease . *Hum Vaccin Immunother* 2014;**10**(6):1478–93. Doi: 10.4161/hv.29054.

28 O. Rossi, M. Caboni, A. Negrea, F. Necchi, R. Alfini, F. Micoli, et al. Toll-Like Receptor

Activation by Generalized Modules for Membrane Antigens from Lipid A Mutants of Salmonella enterica Serovars Typhimurium and Enteritidis. *Clin Vaccine Immunol* 2016;**23**(4):304–14. Doi: 10.1128/CVI.00023-16 LK

29 Meloni Eleonora, Colucci Anna Maria, Micoli Francesca, Sollai Luigi, Gavini Massimiliano, Saul Allan, et al. Simplified low-cost production of O-antigen from Salmonella Typhimurium Generalized Modules for Membrane Antigens (GMMA). *J Biotechnol* 2015;**198**:46–52. Doi: 10.1016/j.jbiotec.2015.01.020.

30 Launay Odile, Lewis David J.M., Anemona Alessandra, Loulergue Pierre, Leahy Jo, Sciré Antonella Silvia, et al. Safety Profile and Immunologic Responses of a Novel Vaccine Against Shigella sonnei Administered Intramuscularly, Intradermally and Intranasally: Results From Two Parallel Randomized Phase 1 Clinical Studies in Healthy Adult Volunteers in Europe. *EBioMedicine* 2017;**22**:164–72. Doi: 10.1016/j.ebiom.2017.07.013.

31 Launay Odile, Ndiaye Augustin G.W., Conti Valentino, Loulergue Pierre, Sciré Antonella Silvia, Landre Anais Maugard, et al. Booster vaccination with GVGH shigella sonnei 1790GAHB GMMA vaccine compared to single vaccination in unvaccinated healthy european adults: Results from a phase 1 clinical trial. *Front Immunol* 2019;**10**(MAR):1–10. Doi: 10.3389/fimmu.2019.00335.

32 Obiero Christina W., Ndiaye Augustin G.W., Sciré Antonella Silvia, Kaunyangi Bonface M., Marchetti Elisa, Gone Ann M., et al. A phase 2a randomized study to evaluate the safety and immunogenicity of the 1790GAHB generalized modules for membrane antigen vaccine against Shigella sonnei administered intramuscularly to adults from a shigellosis-endemic country. *Front Immunol* 2017;**8**(DEC):1–11. Doi: 10.3389/fimmu.2017.01884.

33 Maggiore Luana, Yu Lu, Omasits Ulrich, Rossi Omar, Dougan Gordon, Thomson Nicholas R, et al. Quantitative proteomic analysis of Shigella flexneri and Shigella sonnei Generalized Modules for Membrane Antigens (GMMA) reveals highly pure preparations. *Int J Med Microbiol* 2016;**306**(2):99–108. Doi: 10.1016/j.ijmm.2015.12.003.

34 Micoli Francesca, Rondini Simona, Alfini Renzo, Lanzilao Luisa, Necchi Francesca, Negrea Aurel, et al. Comparative immunogenicity and efficacy of equivalent outer membrane vesicle and glycoconjugate vaccines against nontyphoidal Salmonella . *Proc Natl Acad Sci* 2018;**115**(41):10428–33. Doi: 10.1073/pnas.1807655115.

Hanumunthadu B, *et al. BMJ Open* 2023; 13:e072938. doi: 10.1136/bmjopen-2023-072938

Date and version No: 13January2023 Version 3.1

APPENDIX A: GRADING THE SEVERITY OF SOLICITED AND UNSOLICITED ADVERSE EVENTS

Adverse event	Grade	Definition (in degrees Celsius)
**Temperature**	0	< 37.6
	1	37.6 – 38.0
	2	38.1 – 39.0
	3	> 39

Adverse event	Grade	Definition
**Any symptom**	0	Absence or resolution of symptom
	1	Awareness of symptom but tolerated; transient or mild discomfort; little or no medical intervention required
	2	Discomfort enough to cause limitation of usual activity; some medical intervention or therapy required
	3	Significant interference with daily activity
	4	Emergency department visit or hospitalisation
	5*	Fatality

*All grade 5 AE will be considered either a SAE, SAR, or SUSAR dependant on causality and 'expectedness'

Hanumunthadu B, *et al. BMJ Open* 2023; 13:e072938. doi: 10.1136/bmjopen-2023-072938

Date and version No: 13January2023 Version 3.1

APPENDIX B: GRADING THE SEVERITY OF VISIT OBSERVED ADVERSE EVENTS

Observation	Grade 1	Grade 2	Grade 3
**Oral temperature (ºC)**	37.6 – 38.0	38.1 – 39.0	>39
**Tachycardia (beats/min)**	101-115	116-130	>130
**Bradycardia (beats/min)**	50-54	45-49	<45
**Systolic hyper-tension (mmHg)**	141-150	151-155	>155
**Diastolic hyper-tension (mmHg)**	91-95	96-100	>100
**Systolic hypo-tension (mmHg)**	85-89	80-84	<80

The following ranges are considered normal physiological ranges and are recorded as Grade 0:

- Oral temperature between 35.5 and 37.5 C
- Resting heart rate between 55 and 100 beats/minute
- Systolic blood pressure between 90 and 140 mmHg

Hanumunthadu B, *et al. BMJ Open* 2023; 13:e072938. doi: 10.1136/bmjopen-2023-072938

Date and version No: 13January2023 Version 3.1

APPENDIX C: GRADING THE SEVERITY OF LABORATORY OBSERVED ADVERSE EVENTS

Parameter	Grade 1	Grade 2	Grade 3	Grade 4*
**Haemoglobin: decrease from baseline value (g/l)**	≤ 15	16-20	21-50	>50
**White cell count: elevated (10^9/L)**	11.5–15	>15–20	>20–25	>25
**White cell count: depressed (10^9/L)**	2.5-3.5	1.5-2.49	1.0-1.49	<1.0
**Neutrophil count (10^9/L)**	1.5-1.99	1.0-1.49	0.5-0.99	<0.50
**Platelets (10^9/L)**	125-140	100-124	25-99	<25
**Sodium: hyponatraemia (mmol/L)**	132–134	130–131	125–129	<125
**Sodium: hypernatraemia (mmol/L)**	146	147	148–150	>150
**Potassium: hyperkalaemia (mmol/L)**	5.1–5.2	5.3–5.4	5.5–5.6	>5.6
**Potassium: hypokalaemia (mmol/L)**	3.3–3.4	3.1–3.2	3.0	<3.0
**Urea (mmol/L)**	8.2–8.9	9.0–11	>11	RRT
**Creatinine (µmol/L)**	114-156	157-312	>312	RRT
**ALT and/or AST (IU/L)**	1.1–2.5 x ULN	>2.5–5.0 x ULN	>5.0-10 x ULN	>10 x ULN
**Bilirubin, with increase in LFTs (µmol/L)**	1.1–1.25 x ULN	>1.25–1.5 x ULN	>1.5–1.75 x ULN	>1.75 x ULN
**Bilirubin, with normal LFTs (µmol/L)**	1.1–1.5 x ULN	>1.5–2.0 x ULN	>2.0–3.0 x ULN	>3.0 x ULN
**Alkaline phosphatase (IU/L)**	1.1–2.0 x ULN	>2.0–3.0 x ULN	>3.0–10 x ULN	>10 x ULN
**Albumin: hypoalbuminaemia (g/L)**	28–31	25–27	<25	Not applicable
**C-reactive protein**	>10-30	31-100	101-200	>200

Grade 4* Potentially life threatening

Hanumunthadu B, *et al. BMJ Open* 2023; 13:e072938. doi: 10.1136/bmjopen-2023-072938

Date and version No: 13January2023 Version 3.1

APPENDIX D: WHO CLINICAL PROGRESSION SCALE FOR CLINICAL STUDIES OF COVID-19

Patient State	Descriptor	Score
Uninfected	Uninfected; no viral RNA detected	0
Ambulatory mild disease	Asymptomatic; viral RNA detected	1
	Symptomatic; independent	2
	Symptomatic; assistance needed	3
Hospitalised: moderate disease	Hospitalised; no oxygen therapy*	4
	Hospitalised; oxygen by mask or nasal prongs	5
Hospitalised: severe diseases	Hospitalised; oxygen by NIV or high flow	6
	Intubation and mechanical ventilation, $pO_2/FiO_2 \geq 150$ or $SpO_2/FiO_2 \geq 200$	7
	Mechanical ventilation $pO_2/FIO_2 < 150$ ($SpO_2/FiO_2 < 200$) or vasopressors	8
	Mechanical ventilation $pO_2/FiO_2 < 150$ and vasopressors, dialysis, or ECMO	9
Dead	Dead	10

APPENDIX E: AMENDMENT HISTORY

Amendment No.	Protocol Version No.	Date issued	Author(s) of changes	Details of Changes made
1	3	14/06/2022	Brama Hanumunthadu/Nelly Owino/Maheshi Ramasamy	• Edit to Typhoid / Paratyphoid vaccine as an exclusion criteria<br>• Clarification of SARS-COV-2 test as a temporary exclusion criteria<br>• Addition / modification of mailout language and inclusion of use of GP databases to identify potential participants
NSA01	3.1	13/01/2023	Timothy Crocker-Buque	• The protocol has been has been edited to clarify:<br>• Minor edit to Participant study windows for the V5, V8 and V11 (D28 post vaccination Visit). Edit changed from D28 +/- 4 days to +/- 7 days. This change has been made to improve participant management and Data collection.

List details of all protocol amendments here whenever a new version of the protocol is produced. This is not necessary prior to initial REC / MHRA / HRA submission.
Protocol amendments must be submitted to the Sponsor for approval prior to submission to the REC committee, HRA (where required) or MHRA.

Hanumunthadu B, *et al. BMJ Open* 2023; 13:e072938. doi: 10.1136/bmjopen-2023-072938
