# Supplementary material for: Salmonella Vaccine Study in Oxford (SALVO) trial: protocol for an observer-participant blind randomised placebo-controlled trial of the iNTS-GMMA vaccine within a European cohort
Source: BMJ Open. 2023 Nov 14;13(11):e072938. doi: 10.1136/bmjopen-2023-072938 (PMC10649500; doi:10.1136/bmjopen-2023-072938)
Supplement: Supplementary data [file bmjopen-2023-072938supp002.pdf]

Oxford Vaccine Group
University of Oxford
Centre for Clinical Vaccinology and Tropical Medicine,
Churchill Hospital, Headington, Oxford OX3 7LE
Tel: 01865 611400 info@ovg.ox.ac.uk
www.ovg.ox.ac.uk

Salmonella Vaccine Study in Oxford
SALVO

INFORMED CONSENT FORM

Participant's Name: ____

Participant Initials: _________

Participant Number: S A L — [ ][ ][ ]

If you agree, please initial box:

Section 1: Study Procedures	
1. I confirm that I have read the information sheet dated................... (version............) for this study. I have had the opportunity to consider the information, ask questions and have had these answered satisfactorily.	
2. I have spoken with Dr/Nurse____	
3. I understand that my participation is voluntary and that I am free to withdraw at any time without giving any reason, without my medical care or legal rights being affected.	
4. I have received detailed information about the intervention schedule, study procedures, potential side effects and their importance.	
5. I agree to be randomised to receive either the iNTS-GMMA vaccine (Lower or Full Dose Schedule) or the placebo schedule as detailed in the participant information sheet and I am aware of the risks and side effects associated with each intervention. I am aware that each schedule includes 3 vaccine or placebo administrations.	

6. I will bring the 24-hour contact reply slip, signed by my 24-hour contact prior to receiving the first dose of vaccine or placebo. I agree that the study team may contact this person if I cannot be contacted during the study.	
7. I agree to refrain from donating blood/blood products for the duration of the study.	
8. ***Women only***: I understand the need to ensure that I or my partner use effective contraception one month prior to first vaccination and continue to do so for the remainder of the study.	
**Section 2: Personal Information**	
9. I agree to OVG storing and using my personal information as described in the information booklet.	
10. I agree to my General Practitioner being informed of my participation in this study. I agree to my GP and/or other treating doctors being approached for additional information regarding my medical and vaccination history and study staff to access my NHS medical records either via my GP or the electronic patient records system.	
11. I understand that relevant sections of my medical notes and data collected during the study may be looked at by individuals from University of Oxford (Sponsor), from regulatory authorities [and from the NHS Trust(s)], where it is relevant to my taking part in this research. I give permission for these individuals to have access to my records. I agree to my National Insurance (if UK citizen) or Passport number being used to register me on TOPS. I understand that it will be stored electronically for the duration of the study.	
12. I understand TOPS is a Health Research Authority database that aims to prevent healthy volunteers from taking part in too many studies. I understand that only staff at OVG and other research units can use the database and OVG may call other units, or OVG may be called, to check volunteer details.	
13. I agree to provide my bank account details including my account name, sort code and account number for reimbursement purposes. I understand that my banking details will be stored electronically as described in the information booklet. I understand that my personal information will be shared to the extent required to process or verify eligibility of payments as described in the information booklet.	
**Section 3: Research Samples and Data**	
14. I agree to donate blood and saliva samples. I consider these samples a gift to the University of Oxford and I understand I will not gain any direct personal or financial benefit from them.	

Hanumunthadu B, *et al. BMJ Open* 2023; 13:e072938. doi: 10.1136/bmjopen-2023-072938

15. I agree to my de-identified data and biological samples being sent and stored within and outside of the European Union for analysis by collaborating research groups as described in the information booklet.		
16. I understand and agree that some of my samples will be used to investigate the genetic factors determining the response to iNTS-GMMA vaccine or placebo.		
**If all of the applicable sentences above are initialled, meaning "yes", then please continue:**		
17. I agree to take part in this study.		
**Optional:**		
18. I agree to donate stool samples for this study. I consider these samples a gift to the University of Oxford and I understand I will not gain any direct personal or financial benefit from them.	Yes	No
19. I agree to be contacted about ethically approved research studies for which I may be suitable. I understand that agreeing to be contacted does not oblige me to participate in any further studies.	Yes	No

________________________ ________________ __________________________

Name of Participant *Date* *Signature*

________________________ ________________ __________________________

Name of Person taking Consent *Date* *Signature*

**1 copy for participant; 1 original for researcher site file.*

Hanumunthadu B, *et al. BMJ Open* 2023; 13:e072938. doi: 10.1136/bmjopen-2023-072938
