# Supplementary material for: Salmonella Vaccine Study in Oxford (SALVO) trial: protocol for an observer-participant blind randomised placebo-controlled trial of the iNTS-GMMA vaccine within a European cohort
Source: BMJ Open. 2023 Nov 14;13(11):e072938. doi: 10.1136/bmjopen-2023-072938 (PMC10649500; doi:10.1136/bmjopen-2023-072938)
Supplement: Supplementary data [file bmjopen-2023-072938supp003.pdf]

OXFORD VACCINE GROUP

***Sal**monella* **V**accine Study in **O**xford
SALVO

PARTICIPANT INFORMATION SHEET

You are invited to take part in a study to test a new vaccine against Invasive Non-Typhoidal *Salmonella* (iNTS), an important cause of blood poisoning in children and adults in sub-Saharan Africa. The study is being run by the Oxford Vaccine Group, which is part of the University of Oxford.

Participation in the study is entirely voluntary. Before you decide whether to take part, it is important for you to understand what the study is about and what participation would involve. Please take time to read the information carefully and discuss with others if you wish. If anything is unclear or you would like further information, please contact the study team.

Thank you for taking the time to consider taking part in this study.

Contact Details
Oxford Vaccine Group
Centre for Clinical Vaccinology and Tropical Medicine (CCVTM)
Churchill Hospital
Oxford OX3 7LE
Tel: 01865 611400
Email: info@ovg.ox.ac.uk
Website: www.ovg.ox.ac.uk

Hanumunthadu B, *et al. BMJ Open* 2023; 13:e072938. doi: 10.1136/bmjopen-2023-072938

Who are the Oxford Vaccine Group?

The Oxford Vaccine Group, which is part of the **University of Oxford**, is an independent research team of doctors, nurses and play assistants. We carry out research studies of new and improved vaccines for babies, young children, teenagers and adults, and teach doctors and nurses about immunisations. In the past 5 years alone, over 7,000 participants in the Thames Valley area have taken part in our research studies.

What is invasive Non-Typhoidal *Salmonella* disease?

Non-typhoidal *Salmonellae* are a group of bacteria, that are well known to cause food poisoning throughout the world. However, in certain circumstances they can cause a more serious disease, where *Salmonella* can spread beyond the gut leading to blood poisoning, and in some cases sepsis and death. This is called invasive non-typhoidal *Salmonella* (iNTS) disease and is of particular concern in individuals with a weaker immune system. iNTS disease occurs in over half a million people a year, and particularly affects children under 5 years of age in sub-Saharan Africa, where it causes significant disease with over 200,000 cases and 31,000 deaths per year. Adults can also be affected by iNTS disease, particularly those with HIV, malaria or malnutrition.

What is the purpose of the study?

In this study we are investigating a new vaccine against iNTS called the iNTS-GMMA vaccine. This new vaccine is developed by the GSK Vaccine Institute for Global Health (GVGH), a GlaxoSmithKline (GSK) company based in Italy with which the Oxford Vaccine Group is collaborating for the development of a vaccine against iNTS. This will be the first time this vaccine will be given to human volunteers. It contains small amounts / particles of the outer surface of the two most common bacteria that cause iNTS disease (*Salmonella* Enteritidis and *Salmonella* Typhimurium). These particles previously known as Generalised Modules for Membrane and Antigens', and currently abbreviated to 'GMMAs', constitute the main component of the vaccine. **The vaccine does not contain *Salmonella* bacteria and therefore cannot cause infection or disease**. It is hoped that these GMMA particles can stimulate the immune system to produce a protective response against iNTS bacteria and thus prevent future blood stream infections by these bacteria. The GMMA particles are diluted in Alhydrogel, a common vaccine component designed to reduce local side effects. The study is being conducted to evaluate the safety of the vaccine and how well it stimulates the immune system against iNTS.

Not everyone will receive the active vaccine, some individuals will receive a placebo (non-active comparison). The placebo contains all the components of the iNTS-GMMA vaccine except for the active GMMA particles i.e. Alhydrogel alone and cannot stimulate the immune system to produce a protective response against iNTS bacteria. Participants will be enrolled sequentially into 3 groups and randomly allocated to receive either the iNTS-GMMA vaccine or the placebo. Randomization means that neither you nor your doctor will choose whether you receive the active vaccine or placebo. In this study, a computer will assign this to individual participants, like flipping a coin. You have an equal chance of receiving the vaccine versus placebo in groups 1 and 2 and have more chances of receiving the vaccine than the placebo in group 3.

Hanumunthadu B, *et al. BMJ Open* 2023; 13:e072938. doi: 10.1136/bmjopen-2023-072938

Why have I been invited?

We are inviting healthy adults aged 18 to 55 years old to take part in this study. We would not want to recruit anyone who has significant health issues, anyone with altered immune function or any females who are pregnant or thinking of starting a family soon. We use various ways to contact potential volunteers, including the Electoral Roll and the National Health Applications and Infrastructure Services (NHAIS) who hold the central NHS patient database (Open Exeter). This database identifies all persons within the local area who are in the appropriate age range. Whilst we do commission the invitation to take part in the study, we do not have access to personal data and are not directly responsible for the mail out. In addition, you may have previously expressed an interest in taking part in studies at the Oxford Vaccine Group.

What should I consider?

We are keen to recruit healthy volunteers who are:

- Willing to take part and able to attend all study visits
- Aged between 18 and 55 years old, inclusive
- Willing to allow us to communicate with their GP to notify them of your participation in the study and to check your medical history
- (Females) Willing to use effective contraception from 1 month prior to vaccination and for the remainder of the study

Do I have to take part?

No. We are looking for volunteers. Should you volunteer and later change your mind (for whatever reason) it is your right to do so, and you would not need to provide an explanation to the study team or anyone else. In addition, your decision to withdraw would not affect any ongoing medical care you are or will be receiving.

Whatever you choose it's important that you are happy with your decision and it is not the role of the study team to decide for you. If you choose to withdraw after the receiving the vaccine, we would stop all research related activities. However, we would like to check that you remain well after receiving the vaccine for your own safety which may or may not include follow up visits / blood tests. We would use the samples and data we have collected from you in our analysis of the study, up until the point you informed us that you wanted to withdraw.

What are the possible benefits of taking part?

There are no clear benefits to you if you take part in this study. However, you would have the knowledge that you played a part in the early stages of developing a new vaccine against a bacteria that causes a significant burden of death and disease, particularly in sub-Saharan Africa and in children under 5 years of age for which there are currently no licensed vaccine.

Are there any possible disadvantages or risks from taking part?

In general, the risks are in relation to the vaccine/placebo, blood and oral fluid sampling. In addition, you would be asked to attend regular visits at the CCVTM. In regards to COVID-19 please see the COVID-19 section below.

Hanumunthadu B, *et al. BMJ Open* 2023; 13:e072938. doi: 10.1136/bmjopen-2023-072938

- **Vaccine**

General Vaccines

Intra-muscular vaccination can commonly cause reactions, although most tend to be minor and only last a few days. These may typically include injection site - discomfort, redness, and swelling. As for all vaccines some volunteers occasionally may feel generally unwell, develop fevers, muscle aches, joint aches, headache, experience loss of appetite, nausea / vomiting, abdominal pain or diarrhoea. Not everyone will experience symptoms and if they do occur, they should resolve after a few days.

Anaphylaxis is a very rare but a potentially life-threatening allergic reaction and may occur after immunisation. All clinical staff are trained in the immediate treatment of anaphylactic reactions including the use of intra-muscular adrenaline. It is for this reason you need to wait at least 60 minutes after each vaccine dose is given, as this would be within the typical time frame should this reaction occur.

iNTS GMMA Vaccine

This study is the first time that the iNTS-GMMA vaccine will be given to human participants. The studies performed in animals prior to moving on to human trials have shown good safety results. In addition, GMMA-based vaccines against other bacteria have been safely used in over 190 volunteers. These vaccines were found to be well-tolerated and safe in the volunteers who received them. Nevertheless, this is a new vaccine and there may be side effects we do not know about. It is important for you to be aware of this. However, we have multiple measures in place to ensure your safety during the trial as outlined below.

Throughout the study, the safety of the participants in all groups will be monitored following vaccination. This will be done by reviewing of symptoms at visits and through the electronic Diary (eDiary). For your safety there will be an independent safety committee consisting of an independent panel of experts known as the Data and Safety Monitoring Committee (DSMC) who will know whether you have received vaccine or placebo in order to periodically monitor the overall safety of the trial. This committee will review the safety data particularly as the trial proceeds between Groups 1, 2 and 3 and would be required to approve progression from lower to full dose active iNTS-GMMA vaccine within the trial. Should there be any concerns this committee has the power to halt the trial for further evaluation. In addition, you will be provided with study team contact details who are available 24/7 should you need to contact us.

We would ask you to provide contact details of a person who would act as a second contact. Only to be used in an emergency or needing to contact you urgently.

- **Placebo**

The placebo (Alhydrogel) is a common component used in many vaccines and would not usually cause any side effects other than the ones known for any general vaccine as listed

Hanumunthadu B, *et al. BMJ Open* 2023; 13:e072938. doi: 10.1136/bmjopen-2023-072938

above. Not everyone will experience side effects and if they do occur, they should resolve after a few days.

- **Blood/Oral Fluid Sampling**

Blood tests can be painful and sometimes leave bruising and/or temporary discomfort, but these all resolve in a very short period of time. Rarely fainting can occur. Oral fluid samples are collected with a cotton swab of the mouth and should not cause any discomfort.

- **Pregnancy**

For females, you should not take part in this trial if you are pregnant or breastfeeding. It is currently unknown whether the vaccine being tested is safe during pregnancy. For this reason, it is important that women use adequate contraception during the study period. Women who are not of childbearing potential (i.e. postmenopausal or permanently sterile due to surgery such as a hysterectomy) will not be required to use contraception. This will be discussed with you at the screening visit. If you were to become pregnant during the trial you must tell us immediately and you will be withdrawn from the trial, although we will ask to follow you up for safety reasons.

Male participants with female partners are not required to use barrier methods for the purposes of contraception, as the risks of vaccine excretion are negligible.

- **COVID-19**

It is difficult to predict the time course of the COVID-19 pandemic. Should further peaks occur during the study, we will implement specific measures to ensure your safety whilst taking part in the study. **The safety of our study participants remains the prime consideration during the trial.** If you have not already received a COVID-19 vaccine and you become eligible (according to UK policy) for the vaccine during the study, we would talk to you about this. If you agreed and if it was possible, we would find a mutually agreeable time for you to receive this vaccination. We would not prevent you from receiving this vaccination. During the study we will follow up-to-date government advice including on advising testing, self-isolation, and personal protective equipment (PPE) as necessary. Should you develop symptoms of COVID-19 or test positive for COVID-19 during the study, we would want to know about this prior to coming to clinic. This may affect the timings of your clinic visits and we would want to assess whether it is safe for you to receive further study vaccinations.

What will happen to me if I decide to take part?

- **Recruitment**

If you express an interest in taking part, a member of the Oxford Vaccine Group will contact you by telephone to discuss the study and answer any questions you may have. If you have accessed the participant information sheet via the online questionnaire you will have already answered initial eligibility questions. We would like to ask you a few more detailed questions to assess your eligibility.

Following this, if you are interested and seem suitable for the study then we would arrange for you to come to our clinic for a screening visit. In addition we would send you a consent

Hanumunthadu B, *et al. BMJ Open* 2023; 13:e072938. doi: 10.1136/bmjopen-2023-072938

form (paper or electronic) giving permission for the study team to access your medical records to obtain this information via the electronic patient records or through your GP. We would then ask you to return a copy of the signed consent form (paper or electronic). A countersigned form will be provided at the screening visit. This consent form is only to allow access to your medical records, and not the consent for enrolment on to the study. If you choose to participate in the study a separate consent will be taken for inclusion into the trial.

- **Screening Visit**

The **purpose of screening tests is to ensure that you are eligible to take part** and that by taking part in the study you are not taking on any extra risk to your health. At the screening visit we would sit with you and go through the study in detail. This visit would provide an opportunity for you to ask any questions you might have about the study and what's involved. You would be allowed as much time as you feel necessary before making any decision on whether to take part.

Is coming to screening a commitment to taking part?

No. It's an opportunity to meet with the study staff and ask questions; you do not need to make a decision there and then.

What if I wish to volunteer?

If you are keen to proceed, we would ask you to sign an **informed consent form**. Only once this is signed would we then start any study procedures.

What are the study procedures at the screening visit, if I decide to volunteer?

We would ask you questions about your health, undertake a physical examination and take a blood sample (of 10ml; approximately two tablespoons). We would also require a urine sample. Blood testing includes HIV and hepatitis B and C tests, as well as screening for anaemia, liver and kidney function. For females, we would perform a pregnancy test on your urine sample.

Demographic data including your name, date of birth, gender, ethnicity and contact details will be collected, if not already recorded at the recruitment stage. Data protection regulation governs how we store and process your data. Please see 'What will happen to my data?' below for further information.

We would also seek your consent to register your name on the 'The Over-volunteering Prevention System' (TOPS) national database. This is designed to guard against the potential for harm that can result from excessive volunteering in clinical trials involving investigational medicinal products and blood donations. This would be done using your National Insurance number or passport number, and all information is kept confidential. More information can be found at http://www.hra.nhs.uk/about-the-hra/our-committees/the-over-volunteering-prevention-system/. If we have not already done so, prior to your screening visit we would also ask for your consent to access your medical records via the electronic patient record system or via your GP to obtain any relevant medical history that may affect your participation in the study. Once the study team have confirmed your suitability for the trial, we would inform you and arrange a date for your

first visit. You would be formally enrolled into the study at the time the first vaccine dose is given.

- **Enrolment**

If you are eligible for the study, you will be enrolled into 1 of 3 groups (outlined below) dependant on your enrolment date:

Group 1	Randomly allocated 1:1 to receive either the lower dose iNTS-GMMA vaccine or placebo. Up to twelve participants will be allocated to this group.
Group 2	Randomly allocated 1:1 to receive either the full dose iNTS-GMMA vaccine or placebo. Up to twelve participants will be allocated to this group.
Group 3	Randomly allocated 2:1 to receive either the full dose iNTS-GMMA vaccine or placebo. Up to eighteen participants will be allocated to this group.

Table 1: Enrolment of Groups 1-3

- **Overview of Study Visits**

Each participant would receive 3 vaccinations with the iNTS-GMMA vaccine or placebo at intervals of 0, 2 and 6 months. In addition, each participant would require blood tests immediately before each vaccination and at specific intervals after each vaccination. The study will require a total of 12 visits over a 12-month period.

A simplified overview of the study is shown in the diagram below:

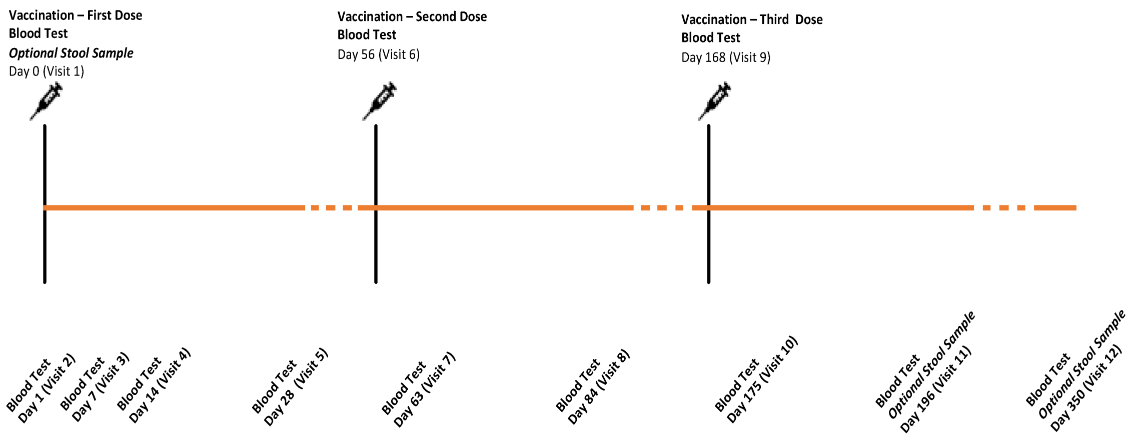

Figure 1: Overview of Study Design

Neither you nor the study team analysing the data will know whether you have received the iNTS-GMMA vaccine or the placebo. This is termed a 'participant-observer blind' trial and provides greater confidence in the final conclusion of the study.

*For participants in group 3 we will collect an additional blood sample (30mls, equivalent to 6 tablespoons) to develop a serum standard at Visit 8 (Day 84). The purpose of the serum

Hanumunthadu B, et al. BMJ Open 2023; 13:e072938. doi: 10.1136/bmjopen-2023-072938

standard is to create a supply of antibodies directed towards the iNTS-GMMA Vaccine that can be used as a reference standard to develop laboratory tests and compare how well this vaccine or future vaccines are working in the individuals being vaccinated. Due to the study design participants who receive the placebo will also donate this additional sample. While these samples will undergo the same laboratory tests, these samples will not be used as a reference standard but may be used in laboratory test development.

- **Vaccine Visits (Visit 1, 6 and 9)**

You would be given a date, time and place to come to the Oxford Vaccine Group at the Churchill Hospital. We would start by checking that you are happy to remain in the study and ask if anything has changed medically since we last saw you.

We would check your pulse and blood pressure at every visit, and for all women a pregnancy test would be done prior to administering each dose of vaccine or placebo. We would take blood and oral samples and give the first vaccine by injection into the muscle of the upper arm.

After vaccination you would need to wait with us for 60 minutes before leaving. This is standard practice to monitor any reactions to the vaccine.

We would give you a tape measure, thermometer and set you up on the **electronic diary** for you to record any symptoms or side effects and daily temperatures for the next **7 days following vaccination**. We would explain how you record this information when you are with us, and it should be entered electronically via a secure link and encrypted transfer on our website wherever possible. Monitoring the ediary will allow the team of research doctors and nurses to check on your wellbeing and ensure your safety.

- **Follow-up Visits**

There are two to four scheduled follow up visits following each vaccination. These visits usually last approximately 30 minutes. We would check on your progress during the trial, review any ediary entries, take recordings of your pulse and blood pressure and collect any blood or oral fluid samples as per the sample schedule below.

- **Samples**

We collect a blood sample at each visit. The amount will be between 5mL (about 1 tablespoon) and 70.5 mL (quarter of a cup) depending on the tests that will be done. The total amount of blood taken during the whole study (1 year) would be up to 667.5mL (about 3 cups), which is significantly less than the maximum amount of blood that could be donated over a year to the UK Blood Donation Service. This is in line with the blood donation guidelines and your body would replace this naturally after about three months. Repeated blood tests can cause anaemia (low haemoglobin), however we have checks and processes in place to ensure this is minimised, including reducing the blood volume we take from you. In rare cases we may ask you to see your general practitioner for a medical review. These blood tests are important to measure how good the vaccine is at producing an immunity to iNTS. In addition, we periodically check your general blood tests including full blood count, urea and electrolytes and liver function tests to ensure they are normal throughout the study.

Hanumunthadu B, *et al. BMJ Open* 2023; 13:e072938. doi: 10.1136/bmjopen-2023-072938

At some visits we will collect an oral fluid sample using a simple 10 swab (1-2 minutes). In addition, to blood we are testing oral fluid to measure indicators of immunity to iNTS.

In addition, stool samples may be collected during the study. This is entirely **optional**. If you do not agree to the collection of stool samples, this will not affect your participation in the study. If you opt-in, the stool samples will be collected before the first vaccine is administered (Visit 1), 28 days after the third vaccine (Visit 11) and at the end of the study (Visit 12). We will test for the microscopic organisms such as bacteria, parasites and fungi that naturally occupy your gut to see if the mix of these microscopic organisms influences your response to the study vaccine. In addition we will test for the development of gut antibodies to Non-typhoidal *Salmonella*e. You will be given collection materials and the study team will explain how to collect the samples. We will receive the samples at the specified visits. However, if you are unable to provide a sample there is an option to free post if you are happy to do so. You will be given 'By Post' collection materials and the study team will explain how to collect and post the sample.

There might be times when your blood needs to be retested or a urine sample taken to confirm test results. This might happen at your next visit or at an unscheduled visit that you would need to come to the clinic for. At this visit the study staff would take a sample of your blood (usually 10 mL, 2 tablespoons) and collect a urine sample, as needed.

What will happen to the samples I give?

Your sample will be assigned a code and your data will also be identified only by this number. The material given to researchers will not have information that directly identifies you. The blood, urine, stool and oral fluid samples collected during this study would be analysed in the Oxford University Hospitals, University of Oxford research laboratories, while some blood samples will be shipped to the GSK Vaccine Institute for Global Health (GVGH), Siena, Italy or delegated laboratories based in Italy. We would also send some samples to other researchers working with us on this research project, including researchers outside the European Union. Your samples will be identified by a code instead of your name and the link between your name and the code number will not be shared with external companies and laboratories.

If you choose to take part in this study, we will be asking for your separate permission to store blood (including cells and DNA) samples, in a collection of samples called Biobank. Details of this will be provided in a separate booklet after you are enrolled into this study, and you are free to say no to the Biobank and continue to take part in this study if you wish. If you do not wish for your samples to be stored in the Biobank, they will be destroyed 12 months after the last participant has completed the study.

What if any of my test results were abnormal?

If there are any abnormal results or undiagnosed conditions found in the course of the study these would be discussed with you and, if you agreed, your GP would be informed of these results. We would not report them to anyone else without your permission. For example, a new diagnosis of high blood pressure might be made. Any newly diagnosed conditions would be looked after by your GP.

Hanumunthadu B, *et al. BMJ Open* 2023; 13:e072938. doi: 10.1136/bmjopen-2023-072938

Reimbursement

There are no costs for you to participate in this study. Study participants would be reimbursed for their time, travel and inconvenience of taking part in the study. The maximum reimbursement for any volunteer who completes the whole study is £585. All participants will be reimbursed based on the following figures:

Travel expenses: £15 per visit
Inconvenience of blood tests: £10 per blood donation
Time required for visits: £20 per visit

The sum reimbursed is on a pro rata basis, so, if for example, you choose to withdraw halfway through the study we would calculate your reimbursement based on the visits you have attended and samples that have been obtained.

Payments are made directly by bank transfer in instalments during the study. For this reason, we would require participants to provide their bank details at screening. Bank details would be kept confidential. Personal information such as your name, bank details and national insurance number may be shared with the University finance team to process or verify your reimbursement payments. Financial auditors may also audit the records where this information is held. All confidential data will be stored according to the UK General Data Protection Regulation (see below).

You may also receive reimbursement for any unscheduled visits you attend (if you have symptoms from the vaccine and need to be assessed). You would be reimbursed £45 per unscheduled visit, up to a maximum of £135 (equivalent of 3 unscheduled visits). If you do not require any unscheduled visits, you will not be reimbursed for this amount.

Would my taking part in this study be kept confidential?

Yes. All information that is collected about you during the course of the research would be coded with a study number and kept strictly confidential. A description of this clinical trial will be available on http://www.ClinicalTrials.gov. This website will not include information that can identify you. You can search this website at any time. The website will include a summary of the research study results, but it may be many years before research results are posted. Any information about you that leaves the clinic would have any identifiable information removed so that you could not be recognized, with the exception of letters sent to your own GP. In order to enrol into this study, you would be required to sign a form, documenting that you consent for us to contact your GP. This is to inform him/her that you would be entering the study, and to ensure there are no medical reasons that would prevent you from taking part in this study. No one else would be told that you are involved in the study. We would only notify your GP of the results from any medical tests we performed with your permission.

Your information would be stored on a secure server, and paper notes would be held by the Oxford Vaccine Group in a locked cabinet. Your data is retained in case we need to contact you regarding any study related matters or if you wish to contact us regarding your participation in the study. We may also contact you to inform you of future related studies.

Responsible members of the University of Oxford and the Oxford University Hospitals NHS Foundation Trust may be given access to data for monitoring and/or audit of the study to ensure that the research is complying with applicable regulations. In addition, the following groups may inspect the study records without violating your confidentiality:

- Monitors who check that the study is being conducted to a high standard, including the Data and Safety Monitoring Committee (DSMC), an independent panel of experts responsible for trial safety and the Medicines and Healthcare Products Regulatory Agency (MHRA).

Coded data and samples would be sent to other researchers working with us on this research project, including researchers outside the European Union. Please note that your blood samples contain cells and DNA. Your DNA is unique to you so it can never be completely anonymous.

What will happen to my data?

Data protection regulation requires that we state the legal basis for processing information about you. In the case of research, this is 'a task in the public interest.' The University of Oxford is the data controller and is responsible for looking after your information and using it properly.

We will be using information from you and your medical records in order to undertake this trial and will use the minimum personally identifiable information possible. We will keep identifiable information about you such as contact details for a minimum of 5 years after the trial has finished. The need to store this information for longer in relation to licensing of the vaccine will be subject to ongoing review.

Paper notes will be held by the Oxford Vaccine Group in a locked cabinet. Once the trial has been completed, all documents, including personally identifiable data, would be archived in a secure facility, for a minimum of 5 years. Storage of this data will be reviewed every 5 years and files will be confidentially destroyed if storage is no longer required. If you complete online or telephone screening, and do not progress to in-person screening, your data will only be stored until the end of the trial.

If you have agreed that samples can be retained for future research then your personally identifiable information will be kept with restricted access solely for the purposes of sample management for a minimum of five years after the last sample has been either used or disposed of in order to meet regulatory requirements. Samples will be provided for future research only in a form that does not identifies you. We store research data securely at the University of Oxford indefinitely following removal of identifiable information.

The trial team will use your name and contact details, to contact you about the clinical trial, and make sure that relevant information about the trial is recorded for your care, in relation to your health during the trial and to oversee the quality of the trial. At the completion of the trial, unless you consent otherwise (e.g. if you request to be informed of other trials),

Hanumunthadu B, *et al. BMJ Open* 2023; 13:e072938. doi: 10.1136/bmjopen-2023-072938

your personal details will not be used to contact you other than exceptional circumstances concerning your safety.

If you consent to take part in another trial carried out by the Oxford Vaccine Centre, we will retain a copy of your consent form until such time as your details are removed from our database but will keep the consent form and your details separate. Personal information and medical information including blood test results may be accessed to avoid unnecessary repetition.

Your bank details will be stored for 7 years in line with university financial policy.
Data protection regulation provides you with control over your personal data and how it is used. When you agree to your information being used in research, however, some of those rights may be limited in order for the research to be reliable and accurate. Professor Andrew J Pollard, or his successor, as Director of the Oxford Vaccine Group will have the responsibility for custody of the data.

Further information about your rights with respect to your personal data is available at: https://compliance.web.ox.ac.uk/individual-rights

If you withdraw from the trial, we will keep the information about you that we have already obtained. To safeguard your rights, we will use the minimum personally identifiable information possible.

You can find out more about how we use your information by contacting Oxford Vaccine Group on 01865 611400 or email info@ovg.ox.ac.uk.

What will happen at the end of the research study?

The results of the research will be published in a scientific medical journal and potentially presented at future conferences; this can potentially take a few years. All OVG publications will appear on the OVG website and you will receive a letter containing these results. Your individual results would not be identifiable, nor would you be identified in any report or publication. The results of the research will also potentially be used for future academic research within the Oxford Vaccine Group. Some of the research being undertaken will also contribute to the fulfilment of an educational requirement (e.g. a doctoral thesis).
Once the last laboratory test is performed in the study, all samples will be destroyed, unless you have consented for them to be transferred to the Biobank. If your samples are going to the Biobank, a copy of your informed consent form (which contains your personal information), are stored with those samples.

What if there is a problem?

If you have private medical insurance, you are advised to contact your insurance company before participating in this trial. The University of Oxford, as Sponsor, has appropriate insurance in place in the unlikely event that you suffer any harm as a direct consequence of your participation in this study.

Hanumunthadu B, *et al. BMJ Open* 2023; 13:e072938. doi: 10.1136/bmjopen-2023-072938

Where can I get advice on whether to take part?

We are happy to answer any questions you might have and contacting us does not commit you to taking part in the study.

Other useful links for general information on taking part in research include:

- www.crn.nihr.ac.uk/can-help/patients-carers-public/how-to-take-part-in-a-study/
- www.nhs.uk/Conditions/Clinical-trials/Pages/Introduction.aspx

What if I wish to complain?

If you wish to complain about any aspect of the way in which you have been approached or treated during the course of this study, you should contact Professor Andrew Pollard, Director of the Oxford Vaccine Group, (Tel: 01865 611400, Email: info@ovg.ox.ac.uk) or you may contact the University of Oxford Research Governance, Ethics and Assurance (RGEA) office on 01865 (6)16480 or the Head of RGEA, email ctrg@admin.ox.ac.uk.

At any time during the study you would be entirely free to change your mind about taking part, and to withdraw from the study. This would not affect your subsequent medical care in any way.

Who is funding the study?

The study is funded by an EU Framework Programme for Research and Innovation, Horizon2020, Vacc-iNTS no 815439 grant, as part of a wider project to progress the iNTS-GMMA vaccine initially through the SALVO clinical trial with a further study to take place in sub-Saharan Africa. For further information on the Vacc-iNTS project please see:
https://vacc-ints.eu

Independent monitoring of the study will be undertaken by Appledown Clinical Research Ltd which will be funded by GSK Vaccine Institute for Global Health (GVGH).

Who has reviewed and approved this study?

All research in the NHS is looked at by an independent group of people, called a Research Ethics Committee, to protect participants' interests. This study has been reviewed and given a favorable opinion by South Central - Oxford A Research Ethics Committee. In addition, this study has been reviewed by the Medicines and Healthcare Regulatory Agency (MHRA) the UK agency responsible for ensuring that medical products under investigation (in this case the active vaccine) is safe and appropriate to continue to clinical trial.

In summary, what would happen if I decide to take part in the study?

- We would ring you to check it is appropriate to include you in the study.
- You would then attend a screening visit in our department (CCVTM).
- At the screening visit we would go through the study in detail and answer any questions you may have. If you are happy to proceed, we would then ask you to sign a consent form. You would then have a brief medical assessment including a medical

history and physical examination. You would have a blood test and urine test (and a pregnancy test for women). These are to assess your eligibility for the study.

- Following satisfactory screening results confirming your eligibility we would invite you to the initial vaccination visit (V1) and enroll you into the study.
- You would be vaccinated with the study vaccine or placebo, once enrolled, then at month two and six.
- You would be seen two to four times after each vaccination; with a final visit one year after the first vaccination. These will include a blood test +/- an oral fluid sample.
- You would be required to fill in an eDiary for seven days after each vaccine.
- The study duration is approximately one year at which time you will be seen 12 times at the CCVTM (not including unscheduled visits or the initial screening visit).

What do I do now?

Thank you for considering taking part in this study. You do not need to make a final decision straight away. If you wish to discuss any element of the study further, then please contact us by either

- telephone **01865 611400**
- website: http://trials.ovg.ox.ac.uk/trials/salvo
- email: info@ovg.ox.ac.uk

Dr Maheshi Ramasamy	Dr Brama Hanumunthadu	Rachel White
Chief Investigator	Lead Doctor	Senior Research Nurse
Consultant Physician		

Hanumunthadu B, *et al. BMJ Open* 2023; 13:e072938. doi: 10.1136/bmjopen-2023-072938
